# Chest pain and shortness of breath in cardiovascular disease: a prospective cohort study in UK primary care

**DOI:** 10.1136/bmjopen-2017-015857

**Published:** 2017-05-25

**Authors:** Lauren A Barnett, James A Prior, Umesh T Kadam, Kelvin P Jordan

**Affiliations:** 1 Research Institute for Primary Care and Health Sciences, Keele University, Staffordshire, UK; 2 Keele Cardiovascular Research Group, Institute for Applied Clinical Sciences, Keele University, Staffordshire, UK

**Keywords:** cardiovascular disease, chest pain, Ischaemic heart disease, Heart failure, shortness of breath

## Abstract

**Objective:**

To determine characteristics associated with monthly chest pain and shortness of breath (SoB) during activity in cardiovascular disease (CVD) and trajectories of these symptoms over 10 months.

**Study design and setting:**

Baseline questionnaire was sent to patients aged ≥40 years from 10 UK general practices. Responders were sent monthly questionnaires for 10 months. For patients with CVD (ischaemic heart disease and heart failure), the association of sociodemographic characteristics, pain elsewhere and anxiety and depression with monthly reports of chest pain and SoB during activity were determined using multilevel, multinomial logistic regression. Common symptom trajectories were determined using dual trajectory latent class growth analysis.

**Results:**

661 patients with CVD completed at least 5 monthly questionnaires. Multiple other pain sites (relative risk ratio: 4.03; 95% CI 1.64 to 9.91) and anxiety or depression (relative risk ratio: 3.31; 95% CI 1.89 to 5.79) were associated with reporting weekly chest pain. Anxiety or depression (relative risk ratio: 4.10; 95% CI 2.72 to 6.17), obesity (relative risk ratio: 2.53; 95% CI 1.49 to 4.30), older age (80+: relative risk ratio: 2.51; 95% CI 1.19 to 5.26), increasing number of pain sites (4+: relative risk ratio: 4.64; 95% CI 2.35 to 9.18) and female gender (relative risk ratio: 1.81; 95% CI 1.20 to 2.75) were associated with reporting weekly SoB. Eight symptom trajectories were identified, with SoB symptoms more common than chest pain.

**Conclusions:**

Potentially modifiable characteristics are associated with the experience of chest pain and SoB. Identified symptom trajectories may facilitate tailored care to improve outcomes in patients with CVD.

**Strengths and limitations of this study**

- This was a large study of primary care patients with diagnosed cardiovascular (CVD) disease.
- It is the first study to examine the course of CVD symptoms based on monthly self-reported data.
- There was attrition, with many patients not completing sufficient monthly questionnaires to be included in the analysis.
- Due to the nature of the questionnaire data, we were unable to identify the number or the duration of the monthly symptom episodes.

## Introduction

Chest pain and shortness of breath (SoB) are common symptoms of cardiovascular disease (CVD) seen in UK primary care. The incidence of chest pain ranges between 13 and 20 per 1000 person-years,^1–3^ with chest pain consultation being a risk factor for future cardiovascular morbidity and mortality.^2 4^ SoB is a frequent symptom of heart failure (HF) and angina.^5 6^ Patients with CVD may experience and consult for chest pain and SoB separately, while many patients will experience both together.^7^

Though directly associated with CVD, chest pain and SoB may also be experienced due to non-cardiac reasons. In a US sample of 10 881 participants with no prevalent CVD or pulmonary conditions, 22% reported SoB,^8^ and SoB and chest pain have been shown to be associated with other patient characteristics such as age, gender and depression.^9^ Understanding potentially modifiable patient characteristics associated with the symptoms of chest pain and SoB (whether related to CVD pathology) in patients diagnosed with CVD may present additional opportunities to direct management to reduce the risk of disease progression and prolonged symptom experience and to improve outcomes in patients with CVD.

Symptoms of chest pain and SoB are not typically isolated events. Assessing how patients’ symptoms co-occur and change over time may help identify common patterns (trajectories) of these symptoms that may be related to long-term outcomes. However, how the experience of chest pain and SoB in patients with CVD varies over time remains unclear. Other trajectories of symptoms have previously been determined to aid understanding of the course of morbidity, for example, in back pain.^10^ Understanding the different trajectories of patient symptoms can be beneficial when trying to optimise healthcare resources, for example, through stratified care.^11^ Such methods may allow better targeting of interventions, potentially earlier or to those with greater need.

The objectives of our study, in a primary care population with CVD, were; (1) to determine the association of patient characteristics with the monthly experience of chest pain or SoB during activity and (2) to determine common trajectories of chest pain and SoB symptoms over time.

## Methods

### Study design

This analysis uses data from the Comorbidity Cohort (2C) study. A prospective cohort study, this was designed to investigate the interaction between CVD and osteoarthritis (OA) comorbidity over 12 months. Full details of the 2C study are reported elsewhere.^12–14^ In brief, the 2C study sampled patients with recorded cardiovascular morbidity and/or OA, aged 40 years or older, plus a reference group without CVD or OA, from the electronic health records of 10 UK general practices. All patients fulfilling the study inclusion criteria were mailed a baseline questionnaire, which included measures of general health as well as CVD and OA specific measures. Those who completed the baseline questionnaire and consented to further follow-up were mailed shorter monthly health questionnaires for the following 10 months and a larger questionnaire at 12 months.

### Study population

This analysis focuses only on those patients with recorded CVD (with or without comorbid OA) of which there were 2858 sent a baseline questionnaire. These patients were identified through a primary care record of ischaemic heart disease (IHD) or HF using Read codes (commonly used to record morbidity in UK primary care) within a 3-year period prior to the baseline questionnaire, starting from November 2006. IHD was identified by Read codes starting G3 (‘Ischaemic heart disease’), and HF was identified by Read codes starting G58 (‘Heart failure’) and codes related to the New York Heart Association classification (codes 662f-i).

### Outcome measures

The primary outcomes were monthly self-reported measures of two CVD-related symptoms: (1) chest pain during activity and (2) SoB during activity. A single-item question was used for each symptom, both based on existing, valid outcome measures (the Seattle Angina Questionnaire^15^ and the Kansas City Cardiomyopathy Questionnaire)^16^. Patients were asked ‘How often (if at all) have you had chest pain during activity over the past 4 weeks?’. The same question structure was used for SoB. Response options for these questions were ‘Not at all’, ‘For 1 week or less’, ‘For 2 weeks’, ‘For 3 weeks’ and ‘For 4 weeks. For analysis, these were categorised into: ‘not at all’, ‘for 1–3 weeks’ and ‘for 4 weeks’, representing no, episodic and persistent monthly symptoms, respectively.

### Covariates

Covariates measured in the baseline questionnaire were age, gender, body mass index (BMI; based on self-reported height and weight), general physical health, pain elsewhere and anxiety and depression. BMI was categorised as: normal weight (<25), overweight (25–30) or obese (30+). General physical health was measured using the 12-item Short-Form Health Survey.^17^ From this, the Physical Component Summary (PCS) score was derived; this is normalised to the US general population with scores below 50 reflecting worse physical health than for the general population. Anxiety and depression were measured using the Hospital Anxiety and Depression Scale with the possible score ranging from 0 (best) to 21 (worst) for anxiety and for depression.^18^ Given the strong association between anxiety and depression, we combined the two scales and defined those with borderline or probable anxiety or depression (based on standard cut-offs) as anxious or depressed. We determined the extent of pain elsewhere at baseline as a count of 10 self-reported areas of pain (neck, shoulder, elbow, hand, back, hip, knee, foot, abdominal and headache). This was categorised into none, 1–3, and 4 or more sites. Lacey *et al*^19^ suggested the number of self-reported pain sites is more important than the pain location.^19^

Baseline chest pain and SoB were measured using yes/no responses to items enquiring about any chest pain or tightness in the past 4 weeks, and any SoB in the past 4 weeks.

### Statistical analyses

The statistical analysis was performed in two phases. The first phase examined associations between baseline sample characteristics and monthly chest pain and SoB experienced during activity. The second phase clustered patients into groups based on the most common patterns (trajectories) of chest pain and SoB experienced over the 10-month follow-up time period. Responders who had completed chest pain or SoB items on at least 5 of the 10 monthly questionnaires were included in the analyses.

#### Phase one: associations between baseline characteristics, chest pain and SoB

A multilevel, multinomial logistic regression model (monthly measurements nested within respondents) was used to determine associations between the baseline sample characteristics (age, gender, BMI, general physical health, pain elsewhere, anxiety and depression) and monthly chest pain during activity (1–3 weeks vs none and 4+ weeks vs none). Associations were initially unadjusted, followed by adjustment for covariates. PCS score was not included in the multivariable model due to strong association with age and pain elsewhere. Interactions between time and covariates that were found to be statistically significant were incorporated into the model to assess whether their effects changed over the time period. The same analysis was then repeated for SoB.

Sensitivity analyses were performed using respondents who completed the chest pain and SoB items on at least 2 monthly questionnaires and by using multiple imputation (with 50 imputations) to impute the missing monthly questionnaire answers and any missing covariate data.

#### Phase two: chest pain and SoB cluster trajectories

Dual trajectory latent class growth analysis (LCGA) was carried out to group (cluster) respondents into the most common trajectories of self-reported chest pain and SoB over the 10 months using the repeated monthly measures of the two symptoms.^20^ Each cluster, therefore, represents a common trajectory of both chest pain and SoB over the 10 months. A fundamental assumption behind dual trajectory LCGA is that the two symptoms, in this case, chest pain and SoB, are correlated. The time order of the measurements were taken into account when deriving the trajectories within a cluster by using quadratic growth curves.

There is no definitive method of deciding on the most appropriate number of clusters using statistical goodness of fit measures. We initially used Akaike’s information criterion, Bayesian information criterion (BIC) and the adjusted BIC, and for each of these, the model with the lowest goodness of fit value indicates the optimal number of clusters. The final decision on the optimal number of clusters was determined by a combination of statistical information, the size and distinctiveness of the clusters and how well the profile (monthly responses of chest pain and SoB) of respondents matched the cluster. Respondents were allocated to the cluster for which they had the highest posterior probability of belonging. The mean of these posterior probabilities for respondents assigned to each cluster (ie, cluster-specific average posterior probabilities (AvePP)) was determined. An AvePP greater than 0.7 suggests clear classification of participants into that cluster.^21^ Cluster-specific probabilities of reporting of chest pain and SoB for each month allow profiles of the trajectory of CVD symptoms to be developed for each cluster. The clusters were then descriptively compared on the baseline covariates described earlier.

As a sensitivity analysis, LCGA was repeated using participants completing the chest pain and SoB items on at least 2 monthly questionnaires. A further sensitivity analysis used multiple imputation to impute clusters for all baseline CVD respondents, with 50 imputations, and using baseline age, gender, BMI, anxiety, depression, PCS score, number of pain sites elsewhere and baseline chest pain, SoB and HF diagnosis in the imputation. All analysis was done using Stata V.14, Mplus V.7.3 and MLwiN V.2.35.

## Results

### Patient demographics

Two thousand and eight hundred and fiftyeight patients with CVD were mailed the baseline questionnaire, of which 1696 (59%) responded. Six hundred and sixty-one (39%) baseline responders answered the chest pain and SoB items on between 5 and 10 monthly questionnaires and were included in the main analyses reported here. Compared with all other baseline responders, those in the analysis were younger (mean difference 2.3 years), had a higher proportion of males (68% vs 62%) and had a slightly lower prevalence of chest pain (42% vs 44%) and SoB (54% vs 58%) at baseline (table 1).

**Table 1:** Sample characteristics, *n* (%)

	Invited at baseline	Baseline responders	At least 1 monthly response	At least 2 monthly responses*	At least 5 monthly responses†
*n*	2858	1696	871	777	661
Condition group					
IHD	2526 (88)	1501 (89)	783 (90)	701 (90)	603 (91)
HF	332 (12)	195 (11)	88 (10)	76 (10)	58 (9)
Age					
<60	401 (14)	172 (10)	103 (12)	89 (11)	75 (11)
60–69	782 (27)	463 (27)	265 (30)	241 (31)	201 (30)
70–79	987 (35)	652 (38)	339 (39)	304 (39)	274 (41)
80+	688 (24)	409 (24)	164 (19)	143 (18)	111 (17)
Gender					
Male	1712 (60)	1059 (62)	574 (66)	514 (66)	447 (68)
Female	1146 (40)	637 (38)	297 (34)	263 (34)	214 (32)
Baseline chest pain	N/A	689 (44)	359 (44)	318 (43)	266 (42)
Baseline SoB	N/A	942 (58)	495 (58)	430 (57)	354 (54)

*Population for sensitivity analysis.

†Population for main analysis.

HF, heart failure; IHD, ischaemic heart disease; SoB, shortness of breath.

During the 10-month follow-up period, prevalence of chest pain during activity in all 4 weeks ranged from 11% to 13% by month and that of chest pain for 1–3 weeks from 23% to 28%. Monthly prevalence of SoB during activity in all 4 weeks ranged from 21% to 26% and SoB for 1–3 weeks from 34% to 37%.

#### Phase one: associations between baseline characteristics and chest pain

Unadjusted analysis showed increasing number of pain sites elsewhere, being anxious or depressed and having a worse PCS score were associated with an increase in risk of reporting chest pain in all 4 weeks in a month.

After adjustment for all covariates, an increasing number of pain sites (relative risk ratio (RRR) for 4+ vs none: 4.03, 95% CI 1.64 to 9.91) and being anxious or depressed (RRR: 3.31, 95% CI 1.89 to 5.79) were associated with reporting chest pain in all 4 weeks in the previous month (table 2). The interactions of anxiety or depression and number of pain sites at baseline with time were not statistically significant.

**Table 2:** Associations between baseline characteristics and monthly chest pain (n=661)

Baseline characteristics	Monthly chest pain			
	1–3 weeks versus none		4 weeks versus none	
	Unadjusted RRR (95% CI)	Adjusted* RRR (95% CI)	Unadjusted RRR (95% CI)	Adjusted* RRR (95% CI)
Male	1	1	1	1
Female	**1.50 (1.08 to 2.08)**	1.20 (0.77 to 1.54)	1.48 (0.90 to 2.43)	1.29 (0.73 to 2.28)
Age				
<60	1	1	1	1
60–69	1.35 (0.81 to 2.23)	1.23 (0.74 to 2.06)	0.64 (0.31 to 1.33)	0.65 (0.28 to 1.49)
70–79	1.39 (0.85 to 2.27)	1.41 (0.86 to 2.34)	0.49 (0.24 to 0.99)	0.49 (0.21 to 1.11)
80+	1.25 (0.68 to 2.29)	1.18 (0.64 to 2.16)	0.42 (0.17 to 1.03)	0.47 (0.17 to 1.28)
BMI				
Normal	1	1	1	1
Overweight	0.88 (0.60 to 1.28)	0.79 (0.54 to 1.15)	0.93 (0.53 to 1.65)	0.80 (0.42 to 1.51)
Obese	0.95 (0.63 to 1.45)	0.67 (0.43 to 1.03)	1.77 (0.94 to 3.34)	0.74 (0.36 to 1.54)
PCS score (per unit)	**0.95 (0.93 to0.96)**	–	**0.89 (0.87 to 0.91)**	–
Not anxious or depressed	1	1	1	1
Anxious or depressed	**2.55 (1.85 to 3.51)**	**2.32 (1.66 to 3.24)**	**5.42 (3.19 to 9.19)**	**3.31 (1.89 to 5.79)**
Pain sites				
None	1	1	1	1
1–3	**3.38 (1.97 to 5.81)**	**3.01 (1.77 to 5.13)**	1.19 (0.50 to 2.81)	1.24 (0.51 to 3.01)
4+	**5.66 (3.32 to 9.66)**	**3.89 (2.25 to6.74)**	**6.16 (2.69 to 14.14)**	**4.03 (1.64 to 9.91)**
Between-person variance	–	2.39	–	7.07

Bold values mean statistically significant

*Adjusted for all presented variables.

BMI, body mass index; PCS, Physical Component Summary; RRR, relative risk ratio.

#### Phase one: associations between baseline characteristics and SoB

Unadjusted analysis showed more pain sites elsewhere, being anxious or depressed, obesity, female gender and having a worse PCS score were associated with an increase in risk of reporting SoB during activity in all 4 weeks in the last month.

After adjustment for all covariates, increasing number of pain sites (RRR for 4+ vs none: 4.64, 95% CI 2.35 to 9.18), being anxious or depressed (RRR: 4.10, 95% CI 2.72 to 6.17) and obesity (RRR: 2.53, 95% CI 1.49 to 4.30) were associated with increased risk of reporting SoB in each of the previous 4 weeks, as were female gender and being over 80 years of age. There were no statistically significant interactions with time (table 3).

**Table 3:** Associations between baseline characteristics and monthly shortness of breath (n=661)

Baseline characteristics	Monthly shortness of breath			
	1–3 weeks versus none		4 weeks versus none	
	Unadjusted RRR (95% CI)	Adjusted* RRR (95% CI)	Unadjusted RRR (95% CI)	Adjusted* RRR (95% CI)
Male	1	1	1	1
Female	**1.84 (1.36 to2.49)**	**1.46 (1.07 to2.01)**	**2.58 (1.78 to 3.75)**	**1.81 (1.20 to 2.75)**
Age				
<60	1	1	1	1
60–69	1.30 (0.81 to 2.07)	1.18 (0.74 to 1.90)	1.10 (0.62 to 1.95)	0.92 (0.49 to 1.71)
70–79	**1.80 (1.14 to 2.83)**	**1.83 (1.15 to2.90)**	1.40 (0.80 to 2.44)	1.72 (0.94 to 3.17)
80+	**1.91 (1.10 to 3.31)**	**2.47 (1.41 to 4.34)**	1.53 (0.77 to 3.02)	**2.51 (1.19 to 5.26)**
BMI				
Normal	1	1	1	1
Overweight	1.25 (0.88 to 1.76)	1.28 (0.90 to 1.81)	1.02 (0.66 to 1.58)	1.03 (0.64 to 1.65)
Obese	**1.77 (1.20 to2.62)**	**1.67 (1.12 to2.51)**	**2.93 (1.80 to4.78)**	**2.53 (1.49 to 4.30)**
PCS score (per unit)	**0.93 (0.92 to0.94)**	–	**0.87 (0.86 to0.89)**	–
Not anxious or depressed	1	1	1	1
Anxious or depressed	**3.04 (2.26 to 4.08)**	**2.33 (1.70 to 3.19)**	**5.36 (3.67 to 7.82)**	**4.10 (2.72 to 6.17)**
Pain sites				
None	1	1	1	1
1–3	**2.23 (1.41 to3.54)**	**1.98 (1.23 to3.17)**	**4.06 (2.20 to7.50)**	**3.50 (1.80 to 6.80)**
4+	**4.31 (2.74 to 6.80)**	**3.05 (1.88 to4.97)**	**8.90 (4.87 to16.29)**	**4.64 (2.35 to 9.18)**
Between-person variance	–	2.09	–	3.72

Bold values mean statistically significant.

*Adjusted for all presented variables.

BMI, body mass index; PCS, Physical Component Summary; RRR, relative risk ratio.

#### Phase one: -sensitivity analyses

Sensitivity analyses including those responding on at least 2 monthly questionnaires (n=777) and using multiple imputation did not change the findings (see online supplementary tables 1–4).

10.1136/bmjopen-2017-015857.supp1 Supplementary tables

### Phase two:- trajectories of chest pain and SoB

The goodness of fit measures from the LCGA did not clearly suggest an optimal number of clusters of trajectories of chest pain and SoB, although their values levelled off from the model with eight clusters (table 4). The six-cluster model had a lowest observed cluster size of 7% (n=47) of participants, compared with 4% (24 participants) for the seven-cluster model, and 3% (20 participants) for the model with eight clusters. Based on the goodness of fit measures, the size of clusters and their interpretation, the eight-cluster model was preferred (table 5).

**Table 4:** Goodness of fit of the dual trajectory models with differing numbers of clusters

Clusters	AIC	BIC	Adjusted BIC	Smallest class (overall) (%)	AvePP* (range)
1	23 241.43	23 277.38	23 251.98		
2	17 999.11	18 066.52	18 018.89		
3	16 185.44	16 284.30	16 214.45	16	0.98–0.98
4	15 088.50	15 218.82	15 126.74	15	0.95–0.99
5	14 310.02	14 471.80	14 357.50	9	0.94–0.99
6	14 018.94	14 212.17	14 075.64	7	0.93–0.98
7	13 820.48	14 045.17	13 886.42	4	0.92–0.98
8	13 701.00	13 957.15	13 776.17	3	0.89–0.99
9	13 628.19	13 915.79	13 712.59	0.1	0.88–1.00

*AvePP for participants allocated to cluster.

AIC, Akaike information criterion; AvePP, average posterior probability; BIC, Bayesian information criterion.

**Table 5:** Summary of the clusters (eight-cluster model)

Description	Cluster 1	Cluster 2	Cluster 3	Cluster 4	Cluster 5	Cluster 6	Cluster 7	Cluster 8
	No symptoms	Infrequent SoB	Occasional chest pain	Occasional SoB	Occasional chest pain and SoB	Frequent chest pain and SoB	Persistent SoB	Persistent chest pain and SoB
Number of patients in cluster (%)	166 (25)	93 (14)	20 (3)	83 (13)	125 (19)	75 (11)	52 (8)	47 (7)
Range of monthly probabilities of extent of chest pain/SoB								
Chest pain								
None	0.95–1.00	0.86–0.91	0.13–0.25	0.85–0.91	0.22–0.27	0.06–0.10	0.85–0.92	<0.01
Up to 3 weeks	<0.01–0.05	0.09–0.14	0.66–0.68	0.09–0.15	0.64–0.67	0.57–0.66	0.08–0.15	0.04–0.10
4 weeks	<0.01	<0.01	0.10–0.20	<0.01	0.09–0.11	0.24–0.37	<0.01	0.90–0.96
SoB								
None	0.96–0.99	0.52–0.65	0.76–0.93	0.13–0.19	0.15–0.16	0.01–0.02	<0.01	<0.01
Up to 3 weeks	0.01–0.04	0.34–0.46	0.07–0.23	0.72–0.74	0.73–0.73	0.33–0.45	0.11–0.16	0.03–0.12
4 weeks	<0.01	0.01–0.02	<0.01	0.09–0.13	0.11–0.12	0.53–0.66	0.84–0.88	0.87–0.97
Median (IQR) % of months with chest pain/SoB								
Chest pain								
None	100 (100,100)	90 (80,100)	21 (0,50)	100 (80,100)	20 (0,40)	0 (0,14)	100 (81,100)	0 (0,0)
Up to 3 weeks	0 (0,0)	10 (0,20)	60 (40,79)	0 (0,20)	70 (56,89)	57 (40,70)	0 (0,13)	0 (0,13)
4 weeks	0 (0,0)	0 (0,0)	0 (0,16)	0 (0,0)	0 (0,11)	33 (17,50)	0 (0,0)	100 (83,100)
SoB								
None	100 (100,100)	63 (50,75)	100 (79,100)	11 (0,25)	10 (0,20)	0 (0,0)	0 (0,0)	0 (0,0)
Up to 3 weeks	0 (0,0)	30 (20,44)	0 (0,17)	80 (70,90)	80 (63,100)	33 (20,50)	0 (0,20)	0 (0,13)
4 weeks	0 (0,0)	0 (0,0)	0 (0,0)	0 (0,20)	0 (0,11)	60 (50,78)	90 (79,100)	100 (86,100)

SoB, shortness of breath.

Patients allocated to the largest cluster (n*=*166, 25%) generally reported no chest pain or SoB symptoms (with monthly probability 0.95 and above) in all the monthly questionnaires. Across clusters, in those reporting symptoms, SoB was more common than chest pain. One cluster of patients reported infrequent breathlessness symptoms (reporting SoB for up to 3 weeks on a median of 3 of the 10 monthly questionnaires: n=93, 14%), another cluster of patients reported occasional breathlessness symptoms (reporting SoB for up to 3 weeks in a median of 8 out of the 10 months: n=83, 13%) and a third cluster of patients reported persistent breathlessness symptoms (reporting SoB in all of the last 4 weeks in all monthly questionnaires: n=52, 8%), but people in these three clusters rarely reported chest pain. The smallest cluster reported occasional chest pain, but rarely reported SoB (n=20, 3%). Two clusters reported occasional (n=125, 19%) or frequent (no months without symptoms, n=75, 11%) pain alongside breathlessness. The most severe group of patients reported persistent SoB and pain over the 10 months, that is, reported SoB and pain for all 4 weeks in all monthly questionnaires (n=47, 7%).

Patients in the clusters with both pain and SoB that was frequent or persistent had poorer baseline comorbid symptoms (anxiety and depression, and pain elsewhere) and worse physical health in general (table 6). Levels of obesity were higher in those with persistent SoB (with or without pain). The most severe group were younger than the other symptomatic groups.

**Table 6:** Comparison between clusters on baseline sociodemographic and general health measures, *n* (%) unless otherwise stated

Description	Cluster 1	Cluster 2	Cluster 3	Cluster 4	Cluster 5	Cluster 6	Cluster 7	Cluster 8
	No symptoms	Infrequent SoB	Occasional chest pain	Occasional SoB	Occasional chest pain and SoB	Frequent chest pain and SoB	Persistent SoB	Persistent chest pain and SoB
Number of patients in cluster	166 (25)	93 (14)	20 (3)	83 (13)	125 (19)	75 (11)	52 (8)	47 (7)
Female	33 (20)	31 (33)	5 (25)	29 (35)	43 (34)	31 (41)	22 (42)	20 (43)
Age								
<60	26 (16)	7 (8)	4 (20)	9 (11)	11 (9)	6 (8)	2 (4)	10 (21)
60–69	59 (36)	28 (30)	5 (25)	17 (20)	42 (34)	17 (23)	17 (33)	16 (34)
70–79	59 (36)	40 (43)	9 (45)	37 (45)	53 (42)	34 (45)	24 (46)	18 (38)
80+	22 (13)	18 (19)	2 (10)	20 (24)	19 (15)	18 (24)	9 (17)	3 (6)
BMI group								
Normal weight	50 (31)	24 (28)	10 (50)	16 (20)	30 (26)	17 (25)	10 (20)	9 (20)
Overweight	81 (50)	47 (55)	7 (35)	39 (49)	57 (49)	29 (43)	16 (31)	14 (32)
Obese	31 (19)	15 (17)	3 (15)	25 (31)	29 (25)	22 (32)	25 (49)	21 (48)
Mean PCS (SD)	47.1 (10.26)	39.5 (10.89)	39.8 (11.4)	38.3 (10.43)	36.93 (9.71)	28.5 (8.91)	31.0 (9.99)	24.6 (7.05)
Anxious or depressed	17 (11)	20 (22)	6 (30)	32 (40)	51 (42)	48 (69)	20 (38)	29 (63)
Pain sites								
None	38 (23)	15 (16)	2 (10)	5 (6)	8 (6)	3 (4)	4 (8)	1 (2)
1–3 sites	83 (50)	39 (42)	8 (40)	30 (36)	45 (36)	19 (25)	25 (48)	10 (21)
4+ sites	45 (27)	39 (42)	10 (50)	48 (58)	72 (58)	53 (71)	23 (44)	36 (77)

BMI, body mas index; PCS, Physical Component Summ ary; SoB, shortness of breath.

Sensitivity analysis, including respondents with at least 2 monthly responses, showed a similar distribution of participants across clusters (see online supplementary table 5). Multiple imputation of cluster membership for all baseline respondents reduced the percentage in the no symptoms cluster (25% to 22%) and increased the percentage in the frequent pain and SoB cluster (11% to 14%) (see online supplementary table 6).

## Discussion

### Summary

Using a prospective cohort study of patients with CVD, we found that potentially modifiable characteristics (weight, psychological health and number of painful body sites) influence the experience of both occasional and persistent chest pain and SoB during activity, and that eight distinct symptom trajectories are apparent, with breathlessness symptoms more common than chest pain.

Three of our identified clusters consisted of patients with stable conditions. The most common cluster (containing a quarter of our sample) reported no symptoms every month. By contrast, one cluster reported persistent chest pain and SoB every month, and another reported persistent SoB without chest pain every month. Symptoms of patients in the remaining clusters fluctuated more. We did not observe a cluster who improved or worsened over the 10 months, which may reflect the prevalent rather than incident nature of the cohort.

Though chest pain and SoB may be considered a distinctive symptom of acute CVD conditions, such as myocardial infarction, a quarter of our prevalent CVD sample did not experience these symptoms at all, similar to a study of community recruited patients with HF from the UK.^22^ Chest pain and SoB commonly co-occurred, but SoB was more common and was reported without chest pain in three of our clusters (containing over a third of our cohort). We found a strong association between poor psychological health and both chest pain and SoB. Our findings are supported by previous cross-sectional studies that showed a correlation between SoB and depression both in US and Chinese secondary care HF samples.^23 24^ Approximately two-thirds of patients with CVD in the most severe symptom clusters (frequent or persistent chest pain and SoB) had anxiety or depression. These results are supported by longitudinal research that reported poorer treatment outcomes in angina patients with chest pain and depression, compared with those without depression^25^ and that in angina patients with persistent symptoms, long-term anxiety and depression were more likely.^26^ Our research shows that the psychological health of patients with CVD, especially those with frequent chest pain and SoB, is poor. However, at present, our findings only indicate the association between chest pain, SoB and poor mental health. It remains unclear whether interventions aimed at anxiety or depression would improve these CVD symptoms, though this does present a potential avenue for future research.

Patients with CVD who were obese at baseline were more likely to experience SoB compared with those of normal weight. This in line with other research and also demonstrates potential to improve this common CVD symptom. Bernhardt and Babb^27^ found that moderate weight loss in women who were obese at baseline was effective at reducing breathlessness during exertion.

We have shown that chest pain and SoB are associated with anxiety and depression and pain elsewhere, and addressing these characteristics may benefit both symptoms. Clark *et al* reported that improving (or diminishing) SoB after 2 years depended on whether pain resolved or diminished respectively.^7^ As a third of our sample reported both chest pain and SoB, these present a considerable proportion of patients with CVD where future research may look to develop interventions towards a singular problem (eg, anxiety), which may also benefit the two symptoms.

Our clusters provide distinct patterns of symptom experience over time within a CVD population. Three-quarters of patients with CVD experienced either SoB or chest pain; however, approximately a third of our sample (35%) reported SoB only, either infrequently (14%), occasionally (13%) or persistently (8%). These may represent a cluster of patients at greater risk of poor outcomes. In a large sample of US participants with coronary artery disease at baseline, the mortality rates were twice as high in patients with SoB compared with those with other symptoms and four times greater than in those with no symptoms at presentation.^28^ Symptoms such a chest pain and SoB may act as prognostic indicators for outcomes resulting in^8^ or from CVD,^28^ and therefore our findings present a range of likely trajectories to which future research may tailor interventions.

### Strengths and limitations

The strength of our study lies in its prospective cohort design that has used a large CVD sample from primary care to gain an insight into the monthly experience of chest pain and SoB symptom in this sample. This study was conducted within a UK primary care setting using monthly surveys from patients with a diagnosis of CVD, identified through electronic health records from 10 general practices. Since these patients were identified from general practices across North Staffordshire, Stoke-on-Trent and Cheshire, they represent a sample generalisable to the UK primary care population.^29^

This is the first research to examine monthly trajectories of these symptoms in CVD patients.

There are several limitations to our work. First, due to the prospective nature of our research, there was attrition during the follow-up period. Those in the monthly analysis were slightly younger and more likely to be male than all those responding at baseline. Related to attrition, our use of multiple imputation may be limited as we are unable to determine if missing values are truly missing at random. In the monthly questionnaire, our definitions of chest pain and SoB were based on a single question for each. Though using a short monthly questionnaire improved the month-on-month response rates, such a simple definition may limit the accuracy of patient responses. The questions related to chest pain and SoB that occurred during activity; however, it is possible that the symptoms may be non-cardiovascular in origin. We also did not identify the number or duration of symptom episodes other than in how many weeks they had occurred in the last month. However, no matter the specific cause of the chest pain or SoB, patients with CVD are experiencing these symptoms, and therefore these need to be addressed.

### Conclusions

Several modifiable patient characteristics (weight, psychological health and number of other painful body sites) are associated with the experience of both occasional and weekly chest pain and SoB during activity. Future interventions to improve CVD symptom experience may wish to target these covariates. By identifying trajectories of symptoms over time, care may be better tailored to improve outcomes in those patients with CVD at greatest risk of worse symptoms and disease progression.

## Supplementary Material

Reviewer comments

Author's manuscript

## References

[1] Walters K, Rait G, Hardoon S, et al Socio-demographic variation in chest pain incidence and subsequent coronary heart disease in primary care in the United Kingdom. Eur J Prev Cardiol 2014;21:566–75. 10.1177/2047487312449415 22617118

[2] Ruigómez A, Rodríguez LA, Wallander MA, et al Chest pain in general practice: incidence, comorbidity and mortality. Fam Pract 2006;23:167–74. 10.1093/fampra/cmi124 16461444

[3] Ruigómez A, Massó-González EL, Johansson S, et al Chest pain without established ischaemic heart disease in primary care patients: associated comorbidities and mortality. Br J Gen Pract 2009;59:78–86. 10.3399/bjgp09X407054 PMC2648936 19275827

[4] Robson J, Ayerbe L, Mathur R, et al Clinical value of chest pain presentation and prodromes on the assessment of cardiovascular disease: a cohort study. BMJ Open 2015;5:e007251 10.1136/bmjopen-2014-007251 PMC4401860 25877275

[5] van Riet EE, Hoes AW, Limburg A, et al Prevalence of unrecognized heart failure in older persons with shortness of breath on exertion. Eur J Heart Fail 2014;16:772–7. 10.1002/ejhf.110 24863953

[6] Montalescot G, Sechtem U, Achenbach S, et al 2013 ESC guidelines on the management of stable coronary artery disease: the task force on the management of stable coronary artery disease of the European Society of Cardiology. Eur Heart J 2013;34:2949–3003. 10.1093/eurheartj/eht296 23996286

[7] Clark N, Fan VS, Slatore CG, et al Dyspnea and pain frequently co-occur among Medicare managed care recipients. Ann Am Thorac Soc 2014;11:890–7. 10.1513/AnnalsATS.201310-369OC 24960243 PMC4213996

[8] Santos M, Kitzman DW, Matsushita K, et al Prognostic Importance of Dyspnea for Cardiovascular Outcomes and Mortality in Persons without Prevalent Cardiopulmonary Disease: the Atherosclerosis risk in Communities Study. PLoS One 2016;11:e0165111 10.1371/journal.pone.0165111 27780208 PMC5079579

[9] Lenfant C Chest pain of cardiac and noncardiac origin. Metabolism 2010;59(Suppl 1):S41–6. 10.1016/j.metabol.2010.07.014 20837193

[10] Dunn KM, Campbell P, Jordan KP Long-term trajectories of back pain: cohort study with 7-year follow-up. BMJ Open 2013;3:e003838 10.1136/bmjopen-2013-003838 PMC3863121 24334157

[11] Hill JC, Whitehurst DG, Lewis M, et al Comparison of stratified primary care management for low back pain with current best practice (STarT Back): a randomised controlled trial. Lancet 2011;378:1560–71. 10.1016/S0140-6736(11)60937-9 21963002 PMC3208163

[12] Prior JA, Rushton CA, Jordan KP, et al Comorbidity Cohort (2C) study: cardiovascular disease severity and comorbid osteoarthritis in primary care. BMC Health Serv Res 2012;12:295 10.1186/1472-6963-12-295 22938503 PMC3488312

[13] Prior JA, Jordan KP, Kadam UT Associations between cardiovascular disease severity, osteoarthritis co-morbidity and physical health: a population-based study. Rheumatology 2014;53:1794–802. 10.1093/rheumatology/keu175 24821851

[14] Rushton CA, Kadam UT Impact of non-cardiovascular disease comorbidity on cardiovascular disease symptom severity: a population-based study. Int J Cardiol 2014 175:154–61. 10.1016/j.ijcard.2014.05.001 24856803 PMC4078220

[15] Dougherty CM, Dewhurst T, Nichol WP, et al Comparison of three quality of life instruments in stable angina pectoris: seattle angina questionnaire, short form health survey (SF-36), and quality of life Index-Cardiac version III. J Clin Epidemiol 1998;51:569–75. 9674663 10.1016/s0895-4356(98)00028-6

[16] Spertus J, Peterson E, Conard MW, et al Monitoring clinical changes in patients with heart failure: a comparison of methods. Am Heart J 2005;150:707–15. 10.1016/j.ahj.2004.12.010 16209970

[17] Ware J, Kosinski M, Keller SD A 12-Item Short-Form health survey: construction of scales and preliminary tests of reliability and validity. Med Care 1996;34:220–33. 8628042 10.1097/00005650-199603000-00003

[18] Zigmond AS, Snaith RP The hospital anxiety and depression scale. Acta Psychiatr Scand 1983;67:361–70. 10.1111/j.1600-0447.1983.tb09716.x 6880820

[19] Lacey RJ, Strauss VY, Rathod T, et al Clustering of pain and its associations with health in people aged 50 years and older: cross-sectional results from the North Staffordshire Osteoarthritis Project. BMJ Open 2015;5:e008389 10.1136/bmjopen-2015-008389 PMC4654278 26553828

[20] Jones BL, Nagin DS Advances in Group-Based Trajectory Modeling and an SAS Procedure for estimating Them. Sociol Methods Res 2007;35:542–71. 10.1177/0049124106292364

[21] Clark DB, Jones BL, Wood DS, et al Substance use disorder trajectory classes: diachronic integration of onset age, severity, and course. Addict Behav 2006;31:995–1009. 10.1016/j.addbeh.2006.03.016 16675151

[22] Clark AL, Goode KM Do patients with chronic heart failure have chest pain? Int J Cardiol 2013;167:185–9. 10.1016/j.ijcard.2011.12.042 22243939

[23] Ramasamy R, Hildebrandt T, O'Hea E, et al Psychological and social factors that correlate with dyspnea in heart failure. Psychosomatics 2006;47:430–4. 10.1176/appi.psy.47.5.430 16959932

[24] Fan X, Meng Z The mutual association between depressive symptoms and dyspnea in Chinese patients with chronic heart failure. Eur J Cardiovasc Nurs 2015;14:310–6. 10.1177/1474515114528071 24634388

[25] Hayek SS, Ko YA, Awad M, et al Depression and chest pain in patients with coronary artery disease. Int J Cardiol 2017;230:420–6. 10.1016/j.ijcard.2016.12.091 28041701 PMC5881400

[26] Jespersen L, Abildstrøm SZ, Hvelplund A, et al Persistent angina: highly prevalent and associated with long-term anxiety, depression, low physical functioning, and quality of life in stable angina pectoris. Clin Res Cardiol 2013;102:571–81. 10.1007/s00392-013-0568-z 23636227

[27] Bernhardt V, Babb TG Weight loss reduces dyspnea on exertion in obese women. Respir Physiol Neurobiol 2014;204:86–92. 10.1016/j.resp.2014.09.004 25220695 PMC4254018

[28] Abidov A, Rozanski A, Hachamovitch R, et al Prognostic significance of Dyspnea in Patients referred for cardiac stress testing. N Engl J Med Overseas Ed 2005;353:1889–98. 10.1056/NEJMoa042741 16267320

[29] Jordan KP, Jöud A, Bergknut C, et al International comparisons of the consultation prevalence of musculoskeletal conditions using population-based healthcare data from England and Sweden. Ann Rheum Dis 2014;73:212–8. 10.1136/annrheumdis-2012-202634 23345602 PMC3888586

