# Supplementary material for: Chest pain and shortness of breath in cardiovascular disease: a prospective cohort study in UK primary care
Source: BMJ Open. 2017 May 25;7(5):e015857. doi: 10.1136/bmjopen-2017-015857 (PMC5726088; doi:10.1136/bmjopen-2017-015857)
Supplement: Supplementary tables [file bmjopen-2017-015857supp001.pdf]

Supplementary Table 1: Results from sensitivity analysis of associations with monthly chest pain using respondents that answered 2 or more monthly questionnaires

	1-3 weeks vs none		4 weeks vs none	
Chest pain (*n* = 777)	Unadjusted RRR (95% CI)	Adjusted[a] RRR (95% CI)	Unadjusted RRR (95% CI)	Adjusted[a] RRR (95% CI)
Age: <60	1	1	1	1
60-69	1.37 (0.86, 2.19)	1.23 (0.76, 1.99)	0.70 (0.35, 1.39)	0.67(0.31, 1.46)
70-79	1.48 (0.93, 2.33)	1.43 (0.89, 2.29)	0.52 (0.26, 1.02)	0.52 (0.24, 1.14)
80+	1.26 (0.73, 2.18)	1.26 (0.72, 2.22)	0.38 (0.17, 0.88)	0.45 (0.17, 1.16)
BMI: Normal	1	1	1	1
Overweight	0.84 (0.59, 1.18)	0.76 (0.54, 1.09)	0.98 (0.56, 1.69)	0.78 (0.42, 1.43)
Obese	0.91 (0.62, 1.33)	0.64 (0.42, 0.95)	1.68 (0.92, 3.05)	0.69 (0.35, 1.39)
Male	1	1	1	1
Female	1.44 (1.07, 1.94)	1.16 (0.84, 1.58)	1.29 (0.81, 2.07)	1.17 (0.68, 2.01)
PCS score (per unit)	0.95 (0.94, 0.96)	-	0.90 (0.88, 0.92)	-
Not anxious/depressed	1	1	1	1
Anxious or depressed	2.66 (1.99, 3.56)	2.33 (1.71, 3.17)	4.84 (2.97, 7.89)	3.20 (1.89, 5.40)
Pain sites: None	1	1	1	1
1-3	3.73 (2.25, 6.21)	3.25 (1.95, 5.41)	1.45 (0.64, 3.29)	1.36 (0.57, 3.22)
4+	6.26 (3.79, 10.33)	4.36 (2.58, 7.38)	6.23 (2.82, 13.78)	4.21 (1.76, 10.11)
Between person variance	-	2.29	-	6.99

Supplementary Table 2: Results from sensitivity analysis of associations with monthly SOB using respondents that answered 2 or more monthly questionnaires.

SoB (*n* = 777)		1-3 weeks vs none Unadjusted RRR (95% CI)	1-3 weeks vs none Adjusted[a] RRR (95% CI)	4 weeks vs none Unadjusted RRR (95% CI)	4 weeks vs none Adjusted[a] RRR (95% CI)
Age:	<60	1	1	1	1
	60-69	1.19 (0.77, 1.83)	1.06 (0.69, 1.65)	1.03 (0.60, 1.74)	0.98 (0.56, 1.72)
	70-79	1.62 (1.06, 2.47)	1.75 (1.13, 2.69)	1.24 (0.74, 2.09)	1.73 (0.99, 3.02)
	80+	1.88 (1.13, 3.12)	2.25 (1.34, 3.76)	1.43 (0.77, 2.67)	2.36 (1.22, 4.60)
BMI:	Normal	1	1	1	1
	Overweight	1.12 (0.81, 1.55)	1.17 (0.84, 1.62)	0.99 (0.66, 1.49)	0.96 (0.62, 1.47)
	Obese	1.71 (1.20, 2.45)	1.53 (1.05, 2.22)	2.86 (1.83, 4.48)	2.20 (1.36, 3.54)
Male		1	1	1	1
Female		1.91 (1.45, 2.52)	1.42 (1.06, 1.90)	2.31 (1.64, 3.25)	1.62 (1.11, 2.34)
PCS score (per unit)		0.93 (0.92, 0.94)	-	0.87 (0.86, 0.89)	-
Not anxious/depressed		1	1	1	1
Anxious or depressed		3.09 (2.36, 4.06)	2.46 (1.85, 3.29)	5.38 (3.81, 7.59)	3.84 (2.66, 5.55)
Pain sites:	None	1	1	1	1
	1-3	2.47 (1.59, 3.84)	2.28 (1.46, 3.55)	4.47 (2.48, 8.05)	3.84 (2.07, 7.14)
	4+	5.02 (3.25, 7.74)	3.51 (2.22, 5.56)	10.46 (5.87, 18.63)	5.60 (2.97, 10.56)
Between person variance		-	2.02	-	3.46

Supplementary Table 3**:** Results from multiple imputation analysis of associations with monthly chest pain response

Multiple Imputation	Monthly chest pain			
Baseline characteristics	1-3 weeks *vs.* none		4 weeks *vs.* none	
	Unadjusted RRR (95% CI)	Adjusted[a] RRR (95% CI)	Unadjusted RRR (95% CI)	Adjusted[a] RRR (95% CI)
Age				
<60	1	1	1	1
60-69	1.27 (0.83, 1.94)	1.22 (0.80, 1.87)	0.66 (0.37, 1.19)	0.68 (0.36, 1.31)
70-79	1.40 (0.94, 2.11)	1.34 (0.89, 2.02)	0.59 (0.33, 1.05)	0.60 (0.32, 1.13)
80+	1.32 (0.83, 2.10)	1.31 (0.81, 2.11)	0.44 (0.22, 0.86)	0.49 (0.23, 1.04)
BMI				
Normal	1	1	1	1
Overweight	0.94 (0.70, 1.27)	0.94 (0.69, 1.27)	0.97 (0.64, 1.49)	0.90 (0.57, 1.43)
Obese	0.94 (0.69, 1.28)	0.79 (0.58, 1.10)	1.71 (1.10, 2.65)	1.17 (0.72, 1.91)
Gender				
Male	1	1	1	1
Female	**1.52 (1.18, 1.96)**	1.27 (0.97, 1.67)	1.43 (0.98, 2.08)	1.20 (0.78, 1.85)
Anxiety/depression				
Not anxious or depressed	1	1	1	1
Anxious or depressed	**2.92 (2.28, 3.74)**	**2.48 (1.84, 3.35)**	**4.69 (3.17, 6.94)**	**3.14 (2.09, 4.71)**
Pain sites:				
None	1	1	1	1
1-3	**3.01 (2.01, 4.54)**	**2.87 (1.90, 4.33)**	1.77 (0.81, 3.88)	1.53 (0.71, 3.31)
4+	**4.43 (3.03, 6.48)**	**3.53 (2.37, 5.26)**	**5.89 (3.20, 10.85)**	**3.91 (1.99, 7.68)**
Between person variance	-	6.07	-	128.51

Supplementary Table 4: Results from multiple imputation analysis of associations with monthly SoB response

Multiple Imputation	Monthly SOB			
Baseline characteristics	1-3 weeks *vs.* none		4 weeks *vs.* none	
	Unadjusted RRR (95% CI)	Adjusted[a] RRR (95% CI)	Unadjusted RRR (95% CI)	Adjusted[a] RRR (95% CI)
Age				
<60	1	1	1	1
60-69	1.37 (0.92, 2.05)	1.36 (0.90, 2.06)	1.05 (0.65, 1.69)	1.10 (0.66, 1.82)
70-79	1.79 (1.22, 2.63)	1.91 (1.29, 2.83)	1.34 (0.85, 2.12)	1.65 (1.01, 2.69)
80+	1.92 (1.25, 2.96)	2.33 (1.50, 3.61)	1.36 (0.80, 2.30)	2.12 (1.21, 3.70)
BMI				
Normal	1	1	1	1
Overweight	1.24 (0.95, 1.62)	1.32 (1.00, 1.74)	1.02 (0.74, 1.41)	1.09 (0.77, 1.54)
Obese	1.51 (1.11, 2.06)	1.56 (1.11, 2.20)	2.61 (1.84, 3.70)	2.60 (1.77, 3.82)
Gender				
Male	1	1	**1**	1
Female	**1.75 (1.38, 2.22)**	**1.40 (1.09, 1.80)**	**2.15 (1.61, 2.88)**	1.59 (1.16, 2.18)
Anxiety/depression				
Not anxious or depressed	1	1	1	1
Anxious or depressed	**2.93 (2.25, 3.82)**	**2.49 (1.88, 3.31)**	**5.16 (3.84, 6.94)**	**4.05 (2.89, 5.68)**
Pain sites:				
None	1	1	1	1
1-3	**2.17 (1.52, 3.10)**	**1.99 (1.36, 2.90)**	3.77 (2.24, 6.35)	3.09 (1.78, 5.36)
4+	**3.38 (2.35, 4.86)**	**2.61 (1.75, 3.90)**	6.44 (3.91, 10.63)	**3.93 (2.24, 6.89)**
Between person variance	-	5.08	-	14.80

Supplementary Table 5: Summary of the clusters (8-cluster model) including patients responding to at least 2 monthly questionnaires

Description	Cluster 1<br>No symptoms	Cluster 2<br>Infrequent SOB	Cluster 3<br>Occasional pain	Cluster 4<br>Occasional SOB	Cluster 5<br>Occasional pain & SOB	Cluster 6<br>Frequent pain & SOB	Cluster 7<br>Persistent SOB	Cluster 8<br>Persistent pain & SOB
Observed *n* (%)	189 (24)	106 (14)	20 (3)	89 (11)	161 (21)	96 (12)	66 (8)	50 (6)
Range of monthly probabilities of extent of chest pain / SOB								
Chest pain<br>None	0.95-1.00	0.85-0.90	0.14-0.24	0.85-0.91	0.22-0.25	0.06-0.11	0.83-0.93	<0.01
Up to 3 weeks	<0.01-0.05	0.10-0.14	0.66-0.68	0.09-0.14	0.66-0.68	0.58-0.68	0.07-0.16	0.04-0.11
4 weeks	<0.01	<0.01	0.10-0.20	<0.01	0.09-0.11	0.21-0.36	<0.01	0.89-0.96
SOB<br>None	0.96-0.99	0.51-0.65	0.76-0.93	0.13-0.18	0.14-0.15	0.01-0.02	<0.01	<0.01
Up to 3 weeks	0.01-0.04	0.33-0.47	0.07-0.23	0.73-0.74	0.73-0.74	0.33-0.43	0.11-0.16	0.03-0.11
4 weeks	<0.01	0.01-0.02	<0.01	0.10-0.13	0.11-0.13	0.55-0.66	0.83-0.87	0.88-0.96

SOB: shortness of breath

Supplementary Table 6: Comparison between clusters on baseline socio-demographic and general health measures using multiple imputed data (m=50)

Description	Cluster 1 No symptoms	Cluster 2 Infrequent SOB	Cluster 3 Occasional pain	Cluster 4 Occasional SOB	Cluster 5 Occasional pain & SOB	Cluster 6 Frequent pain & SOB	Cluster 7 Persistent SOB	Cluster 8 Persistent pain & SOB
Observed %	22	14	3	12	18	14	9	8
Female *n* (%)	24	40	30	42	38	43	50	41
Age <60	13	7	21	8	10	6	5	20
60-69	33	26	29	23	29	21	25	32
70-79	37	39	37	40	39	39	41	35
80+	17	28	14	30	22	34	30	13
BMI group								
Normal weight	31	28	51	22	29	29	20	23
Overweight	50	53	34	47	46	40	30	32
Obese	19	18	15	31	25	31	49	44
PCS mean	45.5 (43.9, 47.1)	37.7 (35.6, 39.7)	38.9 (34.8, 42.9)	36.7 (34.6, 38.7)	35.7 (34.0, 37.3)	27.6 (25.9, 29.3)	29.8 (27.4, 32.2)	25.0 (22.8, 27.2)
Pain sites								
None	26	17	13	8	7	5	12	3
1-3 sites	49	44	44	37	35	27	45	20
4 plus sites	25	39	43	55	58	68	43	76
Anxious or depressed	16	35	38	49	52	77	50	73

SOB: shortness of breath. 50 imputations: model included baseline PCS score, anxiety, depression chest pain, SOB, cluster, age, gender, heart failure, number of pain sites
