# Supplementary material for: Chest pain and shortness of breath in cardiovascular disease: a prospective cohort study in UK primary care
Source: BMJ Open. 2017 May 25;7(5):e015857. doi: 10.1136/bmjopen-2017-015857 (PMC5726088; doi:10.1136/bmjopen-2017-015857)
Supplement: Author's manuscript [file bmjopen-2017-015857.draft_revisions.pdf]

BMJ Open

Trajectories of chest pain and shortness of breath in cardiovascular disease: a prospective cohort study

Journal:	*BMJ Open*
Manuscript ID	bmjopen-2017-015857
Article Type:	Research
Date Submitted by the Author:	05-Jan-2017
Complete List of Authors:	Barnett, Lauren; Keele University, Arthritis Research UK Primary Care Centre<br>Prior, James A.; Keele Univ<br>Kadam, Umesh; Keele University, Health Services Research Unit<br>Jordan, Kelvin; Keele University, Arthritis Research UK Primary Care Centre
**Primary Subject Heading**:	Cardiovascular medicine
Secondary Subject Heading:	Epidemiology
Keywords:	cardiovascular disease, chest pain, Ischaemic heart disease < CARDIOLOGY, Heart failure < CARDIOLOGY, shortness of breath

SCHOLARONE™
Manuscripts

Title: Trajectories of chest pain and shortness of breath in cardiovascular disease: a prospective cohort study

Barnett LA, Prior JA, Kadam UT, Jordan KP

Authors

*Lauren A. Barnett, NIHR Research Methods Fellow, MSc[1]

James A. Prior, NIHR School for Primary Care Launching Fellow, PhD[1]

Umesh T. Kadam, Professor in Clinical Epidemiology & Health Services Research, PhD[2]

Kelvin P. Jordan, Professor of Biostatistics, PhD[1]

Affiliation

[1]Research Institute for Primary Care and Health Sciences, Keele University, UK

[2]Keele Cardiovascular Research Group, Institute for Applied Clinical Sciences, Keele University, UK

***Corresponding author:**

Lauren Barnett

Research Institute for Primary Care and Health Sciences,

Keele University, Staffordshire, United Kingdom,

ST5 5BG

E-mail: l.a.barnett@keele.ac.uk

Tel: +44 (0) 1782 734926

For peer review only - http://bmjopen.bmj.com/site/about/guidelines.xhtml

Objective: To determine characteristics associated with monthly chest pain and shortness of breath (SoB) during activity in cardiovascular disease (CVD), and trajectories of these symptoms over ten months.

Study Design and Setting: Baseline questionnaire was sent to patients aged ≥40 years from ten UK general practices. Responders were sent monthly questionnaires for ten months. For patients with CVD (ischaemic heart disease; heart failure), the association of socio-demographic characteristics, pain elsewhere, and anxiety & depression with monthly reports of chest pain and SoB during activity were determined using multilevel multinomial logistic regression. Common symptom trajectories were determined using dual trajectory latent class growth analysis.

Results: 661 patients with CVD completed at least five monthly questionnaires. Multiple other pain sites (relative risk ratio 4.03; 95% CI 1.64, 9.91) and anxiety or depression (3.31; 1.89, 5.79) were associated with reporting weekly chest pain. Anxiety or depression (3.96; 2.64, 5.93), obesity (2.34; 1.39, 3.94) and female gender (1.87; 1.25, 2.82) were associated with reporting weekly SoB. Eight symptom trajectories were identified with SOB symptoms more common than chest pain.

Conclusions: Modifiable characteristics influence the experience of chest pain and SoB. Identified symptom trajectories may facilitate tailored care to improve outcomes in patients with CVD.

Strengths & Limitations

- Main strength is large CVD population from primary care.
- Weakness can be found in loss to follow-up.
- Unable to verify data was missing at random for the use of multiple imputation
- Unable to identify number or duration of symptom episodes.

For peer review only - http://bmjopen.bmj.com/site/about/guidelines.xhtml

1. Introduction

Chest pain and shortness of breath (SoB) are common symptoms of cardiovascular disease (CVD) seen in UK primary care. The incidence of chest pain ranges between 13-20 per 1,000 person years [1-3], with chest pain consultation being a risk factor for future cardiovascular morbidity and mortality [2,4]. Shortness of breath is a frequent symptom of heart failure (HF) and angina [5,6]. Patients with CVD may experience and consult for chest pain and SoB separately, whilst many patients will experience both together [7].

Though directly associated with CVD, chest pain and SoB may also be experienced due to non-cardiac reasons and have been shown to be associated with other patient characteristics such as age, gender and depression [1,8,9]. Understanding potentially modifiable patient characteristics associated with the symptoms of chest pain and SoB (whether related to CVD pathology or not) in patients diagnosed with CVD may present additional opportunities to direct management to reduce the risk of disease progression and prolonged symptom experience, and improve outcomes in patients with CVD.

Symptoms of chest pain and SoB are not typically isolated events. However, how the experience of chest pain and SoB in patients with CVD varies over time remains unclear. Other trajectories of symptoms have previously been determined to aid understanding of the course of morbidity, for example in back pain [10]. Understanding the different trajectories of patient symptoms can be beneficial when trying to optimise health care resources, for example through stratified care [11]. Such methods may allow better targeting of interventions, potentially earlier or to those with greater need.

The objectives of our study, in a primary care population with CVD, were; i) to determine the association of patient characteristics with the monthly experience of

For peer review only - http://bmjopen.bmj.com/site/about/guidelines.xhtml

chest pain or SoB during activity and ii) to determine common trajectories of chest pain and SoB symptoms over time.

2. Methods

2.1. Study design

This analysis uses data from the Comorbidity Cohort (2C) study. A prospective cohort study, this was designed to investigate the interaction between CVD and osteoarthritis (OA) comorbidity over 12 months. Full details of the 2C study are reported elsewhere [12-14]. In brief, the 2C study sampled patients with recorded cardiovascular morbidity and/or OA, aged 40 years or older, plus a reference group without CVD or OA, from the electronic health records of 10 UK general practices, with a total registered population aged 40 years or older of approximately 46,000. All patients fulfilling the study inclusion criteria were mailed a baseline questionnaire, which included measures of general health, as well as CVD and OA specific measures. Those who completed the baseline questionnaire and consented to further follow-up were mailed shorter monthly health questionnaires for the following 10 months and a larger questionnaire at 12 months. Ethics approval for the 2C study was granted by Cheshire Research Ethics Committee (reference number 09/H1017/40).

2.2. Study Population

This analysis focuses only on those patients with recorded CVD (with or without comorbid OA) of which there were 2,858 sent a baseline questionnaire. These patients were identified through a primary care record of ischaemic heart disease (IHD) or heart failure (HF) using Read Codes (commonly used to record morbidity in UK primary care) within a 3-year period prior to the baseline questionnaire, starting from November

For peer review only - http://bmjopen.bmj.com/site/about/guidelines.xhtml

2006. IHD was identified by Read codes starting G3 ("Ischaemic heart disease"), and HF was identified by Read codes starting G58 ("Heart failure") and codes related to the New York Heart Association (NYHA) classification (codes 662f-i).

2.3. Outcome measures

The primary outcomes were monthly self-reported measures of two CVD-related symptoms: i) chest pain and ii) SoB, both during activity. A single item question was used for each symptom, both based on existing, valid outcome measures (Seattle Angina Questionnaire (SAQ) [15] and the Kansas City Cardiomyopathy Questionnaire (KCCQ) [16]). Patients were asked "How often (if at all) have you had chest pain during activity over the past 4 weeks?". The same question structure was used for SoB. Response options for these questions were "Not at all", "For 1 week or less", "For 2 weeks", "For 3 weeks", and "For 4 weeks. For analysis, these were categorised into: "not at all", "for 1-3 weeks", and "for 4 weeks".

2.4. Covariates

Covariates measured in the baseline questionnaire were age, gender, body mass inded (BMI, based on self-reported height and weight), general physical health, pain elsewhere, and anxiety and depression. BMI was categorised as: normal weight (<25), overweight (25-30) or obese (30+). General physical health was measured using the 12-item Short Form Health Survey (SF-12) [17]. From this, the Physical Component Summary (PCS) score was derived; this is normalized to the US general population with scores below 50 reflecting worse physical health than for the general population. Anxiety and depression were measured using the Hospital Anxiety and Depression scale with the possible score ranging from 0 (best) to 21 (worst) for anxiety and for

For peer review only - http://bmjopen.bmj.com/site/about/guidelines.xhtml

depression [18]. Given the strong association between anxiety and depression, we combined the two scales and defined those with borderline or probable anxiety or depression (based on standard cut-offs) as anxious or depressed. We determined the extent of pain elsewhere at baseline as a count of 10 self-reported areas of pain (neck, shoulder, elbow, hand, back, hip, knee, foot, abdominal and headache). This was categorised into none, 1-3, and 4 or more sites. Lacey et al. (2015) suggested the number of self-reported pain sites is more important than pain location [19].

Baseline chest pain and SoB were measured using yes/no responses to items enquiring about any chest pain or tightness in the past 4 weeks, and any shortness of breath in the past 4 weeks.

2.5. Statistical Analyses

The statistical analysis was performed in two phases. The first phase examined associations between baseline characteristics and monthly chest pain and SoB during activity. The second phase determined common trajectories of chest pain and SoB over the 10-month follow-up time-period. Responders who had completed chest pain or SoB items on at least 5 of the 10 monthly questionnaires were included in the analyses.

2.5.1. Phase one – Associations between baseline characteristics, chest pain and SoB

A multilevel multinomial logistic regression model (monthly measurements nested within respondents) was used to determine associations between the baseline covariates and monthly chest pain during activity (1-3 weeks vs. none, and 4+ weeks vs. none) Associations were initially unadjusted, followed by adjustment for covariates. PCS score was not included in the multivariable model due to strong association with age and pain elsewhere. Interactions with time for all significant covariates were

For peer review only - http://bmjopen.bmj.com/site/about/guidelines.xhtml

incorporated to assess whether effects changed over time. The same analysis was then repeated for SoB.

Sensitivity analyses were performed using respondents who completed the chest pain and SoB items on at least 2 monthly questionnaires and by using multiple imputation with 50 imputations to impute the missing monthly questionnaire answers and any missing covariate data.

2.5.2 Phase two –Trajectories of chest pain and SoB

Dual trajectory Latent Class Growth Analysis (LCGA) was carried out to classify groups of respondents based on the trajectories of their self-reported chest pain and SoB over the 10 months using the repeated measures of the two variables [20]. The underlying assumption of LCGA is that the heterogeneity in both chest pain and SoB across people can be summarised by a certain number of clusters with 2 distinct trajectories for each cluster, i.e. one based on chest pain over the 10 months and the other one based on SoB. LCGA takes into account the time-order of the measurements by applying trend structures on trajectories within each cluster. Quadratic growth curves were applied for all clusters within the LCGA model. There is no definitive method of deciding on the most appropriate number of clusters (groups). We initially used Akaike's information criterion (AIC), Bayesian information criterion (BIC), and the adjusted BIC and for each of these, the model with the lowest goodness of fit value indicates the optimal number of clusters. The final decision on the optimal number of clusters was determined by a combination of statistical information, the size and distinctiveness of the clusters, and how well the chest pain and SoB profiles of respondents allocated to each cluster matched that for the cluster as a whole. Respondents were allocated to the cluster for which they had the highest posterior probability of belonging. Cluster-specific average

For peer review only - http://bmjopen.bmj.com/site/about/guidelines.xhtml

posterior probabilities (AvePP) were calculated as the mean of the posterior probabilities of respondents assigned to a cluster. An AvePP greater than 0.7 suggests clear classification of participants into clusters [21]. Cluster- specific probabilities of reporting of chest pain and SoB for each month allow profiles of the trajectory of CVD symptoms to be developed for each cluster. The clusters were then descriptively compared on the baseline covariates.

As a sensitivity analysis, LCGA was repeated using participants completing the chest pain and SoB items on at least 2 monthly questionnaires. A further sensitivity analysis used multiple imputation to impute clusters for all baseline CVD respondents, with 50 imputations, and using baseline age, gender, BMI, anxiety, depression, PCS score, number of pain sites elsewhere and baseline chest pain, SoB and heart failure diagnosis in the imputation. All analysis was done using Stata 14, Mplus version 7.3 and MLwiN 2.35.

3. Results

3.1. Patient demographics

2,858 patients with IHD and HF were mailed the baseline questionnaire, of which 1,696 (59%) responded. 661 (39%) baseline responders answered the chest pain and SoB items on between 5 and 10 monthly questionnaires and were included in the main analyses reported here. Compared to all other baseline responders, those in the analysis were younger (mean difference 2.3 years), had a higher proportion of males (68% vs 62%), and had a slightly lower prevalence of chest pain (42% vs 44%) and SoB (54% vs 58%) at baseline (**Table 1**).

During the 10-month follow-up period, prevalence of chest pain during activity in all 4 weeks ranged from 11% to 13% by month, and that of chest pain for 1 to 3 weeks from

For peer review only - http://bmjopen.bmj.com/site/about/guidelines.xhtml

23% to 28%. Monthly prevalence of SoB during activity in all 4 weeks ranged from 21% to 26%, and SoB for 1-3 weeks from 34% to 37%.

3.2.1 Phase one - Associations between baseline characteristics and chest pain

Unadjusted analysis showed increasing number of pain sites elsewhere, being anxious or depressed, and having a worse PCS score were associated with an increase in risk of reporting chest pain in all 4 weeks in a month.

After adjustment for all covariates, an increasing number of pain sites (relative risk ratio (RRR) for 4+ vs. none: 4.03, 95% CI: 1.64, 9.91) and being anxious or depressed (RRR: 3.31, 95% CI: 1.89, 5.79) were associated with reporting chest pain in all 4 weeks in the previous month (**Table 2**). The interactions of anxiety or depression, and number of pain sites at baseline with time were not statistically significant.

3.2.2 Phase one - Associations between baseline characteristics and SoB

Unadjusted analysis showed more pain sites elsewhere, being anxious or depressed, obesity, female gender, and having a worse PCS score were associated with an increase in risk of reporting SOB during activity in all 4 weeks in the last month.

After adjustment for all covariates, increasing number of pain sites (RRR for 4+ vs. none: 4.64, 95% CI: 2.35, 9.18), being anxious or depressed (RRR: 4.10, 95% CI: 2.72, 6.17), and obesity (RRR: 2.53, 95% CI: 1.49, 4.30) were associated with increased risk of reporting SOB in each of the previous 4 weeks, as were female gender and being aged over 70. There were no statistically significant interactions with time (**Table 3**).

For peer review only - http://bmjopen.bmj.com/site/about/guidelines.xhtml

3.2.3 Phase one - Sensitivity analyses

Sensitivity analyses including those responding on at least 2 monthly questionnaires (*n*=777) and using multiple imputation did not change the findings (**Supplementary Tables 1-4**).

3.3 Phase two – Trajectories of chest pain and SOB

The goodness of fit measures from the LCGA did not clearly suggest an optimal number of clusters of trajectories of chest pain and SOB, although their values levelled off from the model with 8 clusters (**Table 4**). The six-cluster model had a lowest observed cluster size of 7% (*n*=47) of participants, compared to 4% (24 participants) for the seven-cluster model, and 3% (20 participants) for the model with eight clusters. Based on the goodness of fit measures, the size of clusters and their interpretation, the eight-cluster model was preferred (**Table 5**).

The largest cluster (*n*=166, 25%) generally reported no pain or SoB symptoms (with monthly probability 0.95 and above). In those reporting symptoms, SoB was more common than chest pain. One cluster reported infrequent breathlessness symptoms (reporting SoB for up to 3 weeks on a median of 3 of the monthly questionnaire: *n*=93, 14%), another cluster occasional breathlessness symptoms (median 8 months with SoB for up to 3 weeks: *n*=83, 13%) and a third cluster persistent breathlessness symptoms (in all 4 weeks, for all months: *n*=52, 8%), but people in these three clusters rarely reported chest pain. The smallest cluster reported occasional chest pain, but rarely reported SoB (*n*=20, 3%). Two clusters reported occasional (*n*=125, 19%) or frequent (no months without symptoms, *n*= 75, 11%) pain and breathlessness. The most severe group reported persistent SoB and pain over the 10 months (*n*=47, 7%).

For peer review only - http://bmjopen.bmj.com/site/about/guidelines.xhtml

Patients in the clusters with both pain and SoB that was frequent or persistent had poorer baseline comorbid symptoms (anxiety and depression, and pain elsewhere) and worse physical health in general (**Table 6**). Levels of obesity were higher in those with persistent SoB (with or without pain). The most severe group were younger than the other symptomatic groups.

Sensitivity analysis, including respondents with at least 2 monthly responses, showed a similar distribution of people across clusters (**Supplementary Table 5**). Multiple imputation of cluster membership for all baseline respondents reduced the percentage in the no symptoms cluster (25% to 22%) and increased the percentage in the frequent pain and SoB cluster (11% to 14%) (**Supplementary Table 6**).

4 Discussion

4.1 Summary

Using a prospective cohort study of patients with cardiovascular disease, we found that modifiable characteristics (weight, psychological health, and number of painful body sites) influence the experience of both occasional and persistent chest pain and shortness of breath during activity, and that eight distinct symptom trajectories are apparent, with breathlessness symptoms more common than chest pain.

Though chest pain and shortness of breath may be considered a distinctive symptom of acute CVD conditions, such as myocardial infarction, a quarter of our prevalent CVD sample did not experience these symptoms at all, similar to a study of community recruited HF patients from the UK [22]. We found a strong association between poor psychological health and both chest pain and shortness of breath. Our findings are supported by previous cross-sectional studies which showed a correlation between shortness of breath and depression both in US and Chinese secondary care heart failure

For peer review only - http://bmjopen.bmj.com/site/about/guidelines.xhtml

samples [9,23]. Furthermore, approximately two-thirds of patients with CVD in the most severe symptom clusters (frequent or persistent chest pain and SoB) had anxiety or depression. Our research shows that the psychological health of CVD patients, especially those with frequent chest pain and SoB is poor. However, our findings highlight an opportunity to improve physical symptom experience through mental health. In particular, patients identified with both symptoms may prove the most appropriate group for initial targeting of mental health interventions, particularly in those patients who are optimally managed with anti-anginal medications and heart failure therapy.

CVD patients who were obese at baseline were more likely to experience SoB compared to those of normal weight. This in line with other research, and also demonstrates potential to improve this common CVD symptom. Bernhardt and Babb (2014) found that moderate weight loss in women who were obese at baseline was effective at reducing breathlessness during exertion [24].

We have shown that chest pain and SoB are associated with anxiety and depression and pain elsewhere, and addressing these characteristics may benefit both symptoms. Clark et al reported that improving (or diminishing) SoB after two years depended on whether pain resolved or diminished respectively [7]. As a third of our sample reported both chest pain and SoB, these present a considerable proportion of patients with CVD where interventions towards a singular problem (for example, anxiety) may be of benefit to two symptoms.

4.2 Strengths & limitations

The strength of our study lies in its prospective cohort design which has used a large CVD sample from primary care to gain an insight into the monthly experience of chest

For peer review only - http://bmjopen.bmj.com/site/about/guidelines.xhtml

pain and SoB symptom in this sample. This is the first research to examine monthly trajectories of these symptoms in CVD patients.

There are several limitations to our work. Firstly, due to the prospective nature of our research there was attrition during the follow-up period. Those in the monthly analysis were slightly younger and more likely to be male than all those responding at baseline. Related to attrition, our use of multiple imputation may be limited as we are unable to determine if missing values are truly missing at random. In the monthly questionnaire, our definitions of chest pain and SoB were based on a single question for each. Though using a short monthly questionnaire improved the month-on-month response rates, such a simple definition may limit the accuracy of patient responses. The questions related to chest pain and SoB that occurred during activity; however, it is possible that the symptoms may be non-cardiovascular in origin. We also did not identify the number or duration of symptom episodes other than in how many weeks they had occurred in the last month. However, no matter the specific cause of the chest pain or shortness of breath, patients with CVD are experiencing these symptoms and therefore these need to be addressed.

4.3 Conclusions

Several modifiable patient characteristics (weight, psychological health, and number of other painful body sites) influence the experience of both occasional and weekly chest pain and shortness of breath during activity. By identifying trajectories of symptoms over time, care may be better tailored to improve outcomes in those patients with CVD at greatest risk of worse symptoms and disease progression.

For peer review only - http://bmjopen.bmj.com/site/about/guidelines.xhtml

Acknowledgements: We are grateful for the participation of general practice teams and their patients in supporting the 2C study. Acknowledgements are given to the Keele survey, network, administration and management teams who supported the study.

Contribution statement: Guarantor of overall study integrity: KPJ. Study concept & design: KPJ, JAP & UTK. Data collection and interpretation: LAB, KPJ, JAP & UTK. Statistical analysis: LAB & KPJ. Manuscript preparation: LAB, KPJ, JAP & UTK. Final approval of manuscript: LAB, KPJ, JAP & UTK.

Funding: LAB is funded by an NIHR Research Methods Fellowship, JAP is funded by a Launching Fellowship from the NIHR School for Primary Care Research and UTK was funded by a NIHR Post-Doctoral Fellowship (grant no. PAS/PDA/03/07/035). The study sponsors had no role in study design; in the collection, analysis, and interpretation of data; in the writing of the report; and in the decision to submit the paper for publication. The views expressed are those of the authors and not necessarily those of the NHS, the NIHR or the Department of Health.

Competing interests: The authors have no conflicts of interest

For peer review only - http://bmjopen.bmj.com/site/about/guidelines.xhtml

References

[1] Walters K, Rait G, Hardoon S, Kalaitzaki E, Petersen I, Nazareth I. Socio-demographic variation in chest pain incidence and subsequent coronary heart disease in primary care in the United Kingdom. European Journal of Preventive Cardiology 2014;21:566-75.

[2] Ruigómez A, Rodríguez LAG, Wallander M, Johansson S, Jones R. Chest pain in general practice: incidence, comorbidity and mortality. Family Practice 2006;23:167-74.

[3] Ruigomez A, Masso-Gonzalez EL, Johansson S, Wallander MA, Garcia-Rodriguez LA. Chest pain without established ischaemic heart disease in primary care patients: associated comorbidities and mortality. Br J Gen Pract 2009;59:e78-86.

[4] Robson J, Ayerbe L, Mathur R, Addo J, Wragg A. Clinical value of chest pain presentation and prodromes on the assessment of cardiovascular disease: a cohort study. BMJ Open 2015;5:e007251,2014-007251.

[5] van Riet EES, Hoes AW, Limburg A, Landman MAJ, van der Hoeven H, Rutten FH. Prevalence of unrecognized heart failure in older persons with shortness of breath on exertion 2014;16:772-7.

[6] Task Force Members, Montalescot G, Sechtem U, Achenbach S, Andreotti F, Arden C, et al. 2013 ESC guidelines on the management of stable coronary artery disease: the Task Force on the management of stable coronary artery disease of the European Society of Cardiology. Eur Heart J 2013;34:2949-3003.

[7] Clark N, Fan VS, Slatore CG, Locke E, Whitson HE, Nici L, et al. Dyspnea and pain frequently co-occur among Medicare managed care recipients. Ann Am Thorac Soc 2014;11:890-7.

[8] Lenfant C. Chest pain of cardiac and noncardiac origin. Metab Clin Exp 2010;59, Supplement 1:S41-6.

[9] Fan X, Meng Z. The mutual association between depressive symptoms and dyspnea in Chinese patients with chronic heart failure. European Journal of Cardiovascular Nursing 2015;14:310-6.

[10] Dunn KM, Campbell P, Jordan KP. Long-term trajectories of back pain: cohort study with 7-year follow-up. BMJ Open 2013;3.

[11] Hill JC, Whitehurst DG, Lewis M, Bryan S, Dunn KM, Foster NE, et al. Comparison of stratified primary care management for low back pain with current best practice (STarT Back): a randomised controlled trial. Lancet 2011;378:1560-71.

[12] Prior JA, Rushton CA, Jordan KP, Kadam UT. Comorbidity Cohort (2C) study: Cardiovascular disease severity and comorbid osteoarthritis in primary care. BMC Health Serv Res 2012;12:295.

For peer review only - http://bmjopen.bmj.com/site/about/guidelines.xhtml

[13] Prior JA, Jordan KP, Kadam UT. Associations between cardiovascular disease severity, osteoarthritis co-morbidity and physical health: a population-based study. Rheumatology 2014.

[14] Rushton CA, Kadam UT. Impact of non-cardiovascular disease comorbidity on cardiovascular disease symptom severity: A population-based study. Int J Cardiol 2014;175:154-61.

[15] Dougherty CM, Dewhurst T, Nichol WP, Spertus J. Comparison of three quality of life instruments in stable angina pectoris: Seattle Angina Questionnaire, Short Form Health Survey (SF-36), and Quality of Life Index-Cardiac Version III. J Clin Epidemiol 1998;51:569-75.

[16] Spertus J, Peterson E, Conard MW, Heidenreich PA, Krumholz HM, Jones P, et al. Monitoring clinical changes in patients with heart failure: A comparison of methods. American Heart Journal 2005;150:707-15.

[17] Ware J,Jr, Kosinski M, Keller SD. A 12-Item Short-Form Health Survey: construction of scales and preliminary tests of reliability and validity. Med Care 1996;34:220-33.

[18] Zigmond AS, Snaith RP. The hospital anxiety and depression scale. Acta Psychiatr Scand 1983;67:361-70.

[19] Lacey RJ, Strauss VY, Rathod T, Belcher J, Croft PR, Natvig B, et al. Clustering of pain and its associations with health in people aged 50 years and older: cross-sectional results from the North Staffordshire Osteoarthritis Project. BMJ Open 2015;5.

[20] Jones BL, Nagin DS. Advances in Group-Based Trajectory Modeling and an SAS Procedure for Estimating Them. Sociological Methods & Research 2007;35:542-71.

[21] Clark DB, Jones BL, Wood DS, Cornelius JR. Substance use disorder trajectory classes: diachronic integration of onset age, severity, and course. Addict Behav 2006;31:995-1009.

[22] Clark AL, Goode KM. Do patients with chronic heart failure have chest pain?. Int J Cardiol 2013;167:185-9.

[23] Ramasamy R, Hildebrandt T, O'Hea E, Patel M, Clemow L, Freudenberger R, et al. Psychological and Social Factors That Correlate With Dyspnea in Heart Failure. Psychosomatics 2006;47:430-4.

[24] Bernhardt V, Babb TG. Weight loss reduces dyspnea on exertion in obese women. Respir Physiol Neurobiol 2014;204:86-92.

For peer review only - http://bmjopen.bmj.com/site/about/guidelines.xhtml

Table 1: Sample characteristics, *n* (%)

	Invited at baseline	Baseline responders	At least 1 monthly response	At least 2 monthly responses[a]	At least 5 monthly responses[b]
n	2858	1696	871	777	661
Condition Group:					
IHD	2526 (88)	1501 (89)	783 (90)	701 (90)	603 (91)
HF	332 (12)	195 (11)	88 (10)	76 (10)	58 (9)
Age:					
<60	401 (14)	172 (10)	103 (12)	89 (11)	75 (11)
60-69	782 (27)	463 (27)	265 (30)	241 (31)	201 (30)
70-79	987 (35)	652 (38)	339 (39)	304 (39)	274 (41)
80+	688 (24)	409 (24)	164 (19)	143 (18)	111 (17)
Gender:					
Male	1712 (60)	1059 (62)	574 (66)	514 (66)	447 (68)
Female	1146 (40)	637 (38)	297 (34)	263 (34)	214 (32)
Baseline chest pain:	N/A	689 (44)	359 (44)	318 (43)	266 (42)
Baseline SOB:	N/A	942 (58)	495 (58)	430 (57)	354 (54)

[a] population for sensitivity analysis; [b] population for main analysis.

SOB: shortness of breath, IHD: ischaemic heart disease, HF: heart failure

For peer review only - http://bmjopen.bmj.com/site/about/guidelines.xhtml

Table 2: Associations between baseline characteristics and monthly chest pain (n=661)

Baseline characteristics	Monthly chest pain			
	1-3 weeks *vs.* none		4 weeks *vs.* none	
	Unadjusted RRR (95% CI)	Adjusted[a] RRR (95% CI)	Unadjusted RRR (95% CI)	Adjusted[a] RRR (95% CI)
Male	1	1	1	1
Female	**1.50 (1.08, 2.08)**	1.20 (0.77, 1.54)	1.48 (0.90, 2.43)	1.29 (0.73, 2.28)
Age				
<60	1	1	1	1
60-69	1.35 (0.81, 2.23)	1.23 (0.74, 2.06)	0.64 (0.31, 1.33)	0.65 (0.28, 1.49)
70-79	1.39 (0.85, 2.27)	1.41 (0.86, 2.34)	0.49 (0.24, 0.99)	0.49 (0.21, 1.11)
80+	1.25 (0.68, 2.29)	1.18 (0.64, 2.16)	0.42 (0.17, 1.03)	0.47 (0.17, 1.28)
BMI				
Normal	1	1	1	1
Overweight	0.88 (0.60, 1.28)	0.79 (0.54, 1.15)	0.93 (0.53, 1.65)	0.80 (0.42, 1.51)
Obese	0.95 (0.63, 1.45)	0.67 (0.43, 1.03)	1.77 (0.94, 3.34)	0.74 (0.36, 1.54)
PCS score (per unit)	**0.95 (0.93, 0.96)**	-	**0.89 (0.87, 0.91)**	-
Not anxious or depressed	1	1	1	1
Anxious or depressed	**2.55 (1.85, 3.51)**	**2.32 (1.66, 3.24)**	**5.42 (3.19, 9.19)**	**3.31 (1.89, 5.79)**
Pain sites:				
None	1	1	1	1
1-3	**3.38 (1.97, 5.81)**	**3.01 (1.77, 5.13)**	1.19 (0.50, 2.81)	1.24 (0.51, 3.01)
4+	**5.66 (3.32, 9.66)**	**3.89 (2.25, 6.74)**	**6.16 (2.69, 14.14)**	**4.03 (1.64, 9.91)**
Between person Variance	-	2.39	-	7.07

[a] adjusted for all presented variables. RRR: relative risk ratio, CI: confidence interval, BMI: body mass index, PCS: physical component summary score. **Bold** = statistically significant

For peer review only

Table 3: Associations between baseline characteristics and monthly shortness of breath (n=661)

Baseline characteristics	Monthly shortness of breath			
	1-3 weeks *vs.* none		4 weeks *vs.* none	
	Unadjusted RRR (95% CI)	Adjusted[a] RRR (95% CI)	Unadjusted RRR (95% CI)	Adjusted[a] RRR (95% CI)
Male	1	1	1	1
Female	**1.84 (1.36, 2.49)**	**1.46 (1.07, 2.01)**	**2.58 (1.78, 3.75)**	**1.81 (1.20, 2.75)**
Age				
<60	1	1	1	1
60-69	1.30 (0.81, 2.07)	1.18 (0.74, 1.90)	1.10 (0.62, 1.95)	0.92 (0.49, 1.71)
70-79	**1.80 (1.14, 2.83)**	**1.83 (1.15, 2.90)**	1.40 (0.80, 2.44)	1.72 (0.94, 3.17)
80+	**1.91 (1.10, 3.31)**	**2.47 (1.41, 4.34)**	1.53 (0.77, 3.02)	**2.51 (1.19, 5.26)**
BMI				
Normal	1	1	1	1
Overweight	1.25 (0.88, 1.76)	1.28 (0.90, 1.81)	1.02 (0.66, 1.58)	1.03 (0.64, 1.65)
Obese	**1.77 (1.20, 2.62)**	**1.67 (1.12, 2.51)**	**2.93 (1.80, 4.78)**	**2.53 (1.49, 4.30)**
PCS score (per unit)	**0.93 (0.92, 0.94)**	-	**0.87 (0.86, 0.89)**	-
Not anxious or depressed	1	1	1	1
Anxious or depressed	**3.04 (2.26, 4.08)**	**2.33 (1.70, 3.19)**	**5.36 (3.67, 7.82)**	**4.10 (2.72, 6.17)**
Pain sites:				
None	1	1	1	1
1-3	**2.23 (1.41, 3.54)**	**1.98 (1.23, 3.17)**	**4.06 (2.20, 7.50)**	**3.50 (1.80, 6.80)**
4+	**4.31 (2.74, 6.80)**	**3.05 (1.88, 4.97)**	**8.90 (4.87, 16.29)**	**4.64 (2.35,9.18)**
Between person Variance	-	2.09	-	3.72

[a] adjusted for all presented variables. RRR: relative risk ratio, CI: confidence interval, BMI: body mass index, PCS: physical component summary score. **Bold** = statistically significant

For peer review only

For peer review only - http://bmjopen.bmj.com/site/about/guidelines.xhtml

Table 4 : Goodness of fit of the dual trajectory models with differing numbers of clusters

Clusters	AIC	BIC	Adjusted BIC	Smallest class (overall)	AvePP[a] (range)
1	23241.43	23277.38	23251.98		
2	17999.11	18066.52	18018.89		
3	16185.44	16284.30	16214.45	16%	0.98-0.98
4	15088.50	15218.82	15126.74	15%	0.95-0.99
5	14310.02	14471.80	14357.50	9%	0.94-0.99
6	14018.94	14212.17	14075.64	7%	0.93-0.98
7	13820.48	14045.17	13886.42	4%	0.92-0.98
8	13701.00	13957.15	13776.17	3%	0.89-0.99
9	13628.19	13915.79	13712.59	0.1%	0.88-1.00

[a] Average posterior probability for participants allocated to cluster. AIC: Akaike information criterion, BIC: Bayesian information criterion.

For peer review only

Table 5: Summary of the clusters (8-cluster model)

Description	Cluster 1 No Symptoms	Cluster 2 Infrequent SOB	Cluster 3 Occasional chest pain	Cluster 4 Occasional SOB	Cluster 5 Occasional chest pain & SOB	Cluster 6 Frequent chest pain & SOB	Cluster 7 Persistent SOB	Cluster 8 Persistent chest pain & SOB
Observed *n* (%)	166 (25)	93 (14)	20 (3)	83 (13)	125 (19)	75 (11)	52 (8)	47 (7)
**Range of monthly probabilities of extent of chest pain / SOB**								
Chest pain								
None	0.95-1.00	0.86-0.91	0.13-0.25	0.85-0.91	0.22-0.27	0.06-0.10	0.85-0.92	<0.01
Up to 3 weeks	<0.01-0.05	0.09-0.14	0.66-0.68	0.09-0.15	0.64-0.67	0.57-0.66	0.08-0.15	0.04-0.10
4 weeks	<0.01	<0.01	0.10-0.20	<0.01	0.09-0.11	0.24-0.37	<0.01	0.90-0.96
SoB								
None	0.96-0.99	0.52-0.65	0.76-0.93	0.13-0.19	0.15-0.16	0.01-0.02	<0.01	<0.01
Up to 3 weeks	0.01-0.04	0.34-0.46	0.07-0.23	0.72-0.74	0.73-0.73	0.33-0.45	0.11-0.16	0.03-0.12
4 weeks	<0.01	0.01-0.02	<0.01	0.09-0.13	0.11-0.12	0.53-0.66	0.84-0.88	0.87-0.97
**Median (IQR) % of months with chest pain / SOB**								
Chest pain								
None	100 (100,100)	90 (80,100)	21 (0,50)	100 (80,100)	20 (0,40)	0 (0,14)	100 (81,100)	0 (0,0)
Up to 3 weeks	0 (0,0)	10 (0,20)	60 (40,79)	0 (0,20)	70 (56,89)	57 (40,70)	0 (0,13)	0 (0,13)
4 weeks	0 (0,0)	0 (0,0)	0 (0,16)	0 (0,0)	0 (0,11)	33 (17,50)	0 (0,0)	100 (83,100)
SoB								
None	100 (100,100)	63 (50,75)	100 (79,100)	11 (0,25)	10 (0,20)	0 (0,0)	0 (0,0)	0 (0,0)
Up to 3 weeks	0 (0,0)	30 (20,44)	0 (0,17)	80 (70,90)	80 (63,100)	33 (20,50)	0 (0,20)	0 (0,13)
4 weeks	0 (0,0)	0 (0,0)	0 (0,0)	0 (0,20)	0 (0,11)	60 (50,78)	90 (79,100)	100 (86,100)

SoB: shortness of breath

For peer review only

For peer review only - http://bmjopen.bmj.com/site/about/guidelines.xhtml

Table 6: Comparison between clusters on baseline socio-demographic and general health measures, *n* (%) unless otherwise stated

Description	Cluster 1 No symptoms	Cluster 2 Infrequent SOB	Cluster 3 Occasional chest pain	Cluster 4 Occasional SOB	Cluster 5 Occasional chest pain & SOB	Cluster 6 Frequent chest pain & SOB	Cluster 7 Persistent SOB	Cluster 8 Persistent chest pain & SOB
Observed *n*	166	93	20	83	125	75	52	47
Female	33 (20)	31 (33)	5 (25)	29 (35)	43 (34)	31 (41)	22 (42)	20 (43)
Age								
<60	26 (16)	7 (8)	4 (20)	9 (11)	11 (9)	6 (8)	2 (4)	10 (21)
60-69	59 (36)	28 (30)	5 (25)	17 (20)	42 (34)	17 (23)	17 (33)	16 (34)
70-79	59 (36)	40 (43)	9 (45)	37 (45)	53 (42)	34 (45)	24 (46)	18 (38)
80+	22 (13)	18 (19)	2 (10)	20 (24)	19 (15)	18 (24)	9 (17)	3 (6)
BMI group								
Normal weight	50 (31)	24 (28)	10 (50)	16 (20)	30 (26)	17 (25)	10 (20)	9 (20)
Overweight	81 (50)	47 (55)	7 (35)	39 (49)	57 (49)	29 (43)	16 (31)	14 (32)
Obese	31 (19)	15 (17)	3 (15)	25 (31)	29 (25)	22 (32)	25 (49)	21 (48)
Mean PCS (SD)	47.1 (10.26)	39.5 (10.89)	39.8 (11.4)	38.3 (10.43)	36.93 (9.71)	28.5 (8.91)	31.0 (9.99)	24.6 (7.05)
Anxious or depressed	17 (11)	20 (22)	6 (30)	32 (40)	51 (42)	48 (69)	20 (38)	29 (63)
Pain sites								
None	38 (23)	15 (16)	2 (10)	5 (6)	8 (6)	3 (4)	4 (8)	1 (2)
1-3 sites	83 (50)	39 (42)	8 (40)	30 (36)	45 (36)	19 (25)	25 (48)	10 (21)
4 plus sites	45 (27)	39 (42)	10 (50)	48 (58)	72 (58)	53 (71)	23 (44)	36 (77)

SOB: shortness of breath, BMI: body mass index, PCS: physical component summary score, SD: standard deviation

For peer review only - http://bmjopen.bmj.com/site/about/guidelines.xhtml

Supplementary Table 1: Results from sensitivity analysis of associations with monthly chest pain using respondents that answered 2 or more monthly questionnaires

Chest pain (*n* = 777)	1-3 weeks vs none		4 weeks vs none	
	Unadjusted RRR (95% CI)	Adjusted[a] RRR (95% CI)	Unadjusted RRR (95% CI)	Adjusted[a] RRR (95% CI)
Age: <60	1	1	1	1
60-69	1.37 (0.86, 2.19)	1.23 (0.76, 1.99)	0.70 (0.35, 1.39)	0.67(0.31, 1.46)
70-79	1.48 (0.93, 2.33)	1.43 (0.89, 2.29)	0.52 (0.26, 1.02)	0.52 (0.24, 1.14)
80+	1.26 (0.73, 2.18)	1.26 (0.72, 2.22)	0.38 (0.17, 0.88)	0.45 (0.17, 1.16)
BMI: Normal	1	1	1	1
Overweight	0.84 (0.59, 1.18)	0.76 (0.54, 1.09)	0.98 (0.56, 1.69)	0.78 (0.42, 1.43)
Obese	0.91 (0.62, 1.33)	0.64 (0.42, 0.95)	1.68 (0.92, 3.05)	0.69 (0.35, 1.39)
Male	1	1	1	1
Female	1.44 (1.07, 1.94)	1.16 (0.84, 1.58)	1.29 (0.81, 2.07)	1.17 (0.68, 2.01)
PCS score (per unit)	0.95 (0.94, 0.96)	-	0.90 (0.88, 0.92)	-
Not anxious/depressed	1	1	1	1
Anxious or depressed	2.66 (1.99, 3.56)	2.33 (1.71, 3.17)	4.84 (2.97, 7.89)	3.20 (1.89, 5.40)
Pain sites: None	1	1	1	1
1-3	3.73 (2.25, 6.21)	3.25 (1.95, 5.41)	1.45 (0.64, 3.29)	1.36 (0.57, 3.22)
4+	6.26 (3.79, 10.33)	4.36 (2.58, 7.38)	6.23 (2.82, 13.78)	4.21 (1.76, 10.11)
Between person variance	-	2.29	-	6.99

For peer review only

Supplementary Table 2: Results from sensitivity analysis of associations with monthly SOB using respondents that answered 2 or more monthly questionnaires.

		1-3 weeks vs none		4 weeks vs none	
SoB (*n* = 777)		Unadjusted RRR (95% CI)	Adjusted[a] RRR (95% CI)	Unadjusted RRR (95% CI)	Adjusted[a] RRR (95% CI)
Age:	<60	1	1	1	1
	60-69	1.19 (0.77, 1.83)	1.06 (0.69, 1.65)	1.03 (0.60, 1.74)	0.98 (0.56, 1.72)
	70-79	1.62 (1.06, 2.47)	1.75 (1.13, 2.69)	1.24 (0.74, 2.09)	1.73 (0.99, 3.02)
	80+	1.88 (1.13, 3.12)	2.25 (1.34, 3.76)	1.43 (0.77, 2.67)	2.36 (1.22, 4.60)
BMI:	Normal	1	1	1	1
	Overweight	1.12 (0.81, 1.55)	1.17 (0.84, 1.62)	0.99 (0.66, 1.49)	0.96 (0.62, 1.47)
	Obese	1.71 (1.20, 2.45)	1.53 (1.05, 2.22)	2.86 (1.83, 4.48)	2.20 (1.36, 3.54)
Male		1	1	1	1
Female		1.91 (1.45, 2.52)	1.42 (1.06, 1.90)	2.31 (1.64, 3.25)	1.62 (1.11, 2.34)
PCS score (per unit)		0.93 (0.92, 0.94)	-	0.87 (0.86, 0.89)	-
Not anxious/depressed		1	1	1	1
Anxious or depressed		3.09 (2.36, 4.06)	2.46 (1.85, 3.29)	5.38 (3.81, 7.59)	3.84 (2.66, 5.55)
Pain sites:	None	1	1	1	1
	1-3	2.47 (1.59, 3.84)	2.28 (1.46, 3.55)	4.47 (2.48, 8.05)	3.84 (2.07, 7.14)
	4+	5.02 (3.25, 7.74)	3.51 (2.22, 5.56)	10.46 (5.87, 18.63)	5.60 (2.97, 10.56)
Between person variance		-	2.02	-	3.46

For peer review only

Supplementary Table 3: Results from multiple imputation analysis of associations with monthly chest pain response

Multiple Imputation	Monthly chest pain			
Baseline characteristics	1-3 weeks *vs.* none		4 weeks *vs.* none	
	Unadjusted RRR (95% CI)	Adjusted[a] RRR (95% CI)	Unadjusted RRR (95% CI)	Adjusted[a] RRR (95% CI)
Age				
<60	1	1	1	1
60-69	1.27 (0.83, 1.94)	1.22 (0.80, 1.87)	0.66 (0.37, 1.19)	0.68 (0.36, 1.31)
70-79	1.40 (0.94, 2.11)	1.34 (0.89, 2.02)	0.59 (0.33, 1.05)	0.60 (0.32, 1.13)
80+	1.32 (0.83, 2.10)	1.31 (0.81, 2.11)	0.44 (0.22, 0.86)	0.49 (0.23, 1.04)
BMI				
Normal	1	1	1	1
Overweight	0.94 (0.70, 1.27)	0.94 (0.69, 1.27)	0.97 (0.64, 1.49)	0.90 (0.57, 1.43)
Obese	0.94 (0.69, 1.28)	0.79 (0.58, 1.10)	1.71 (1.10, 2.65)	1.17 (0.72, 1.91)
Gender				
Male	1	1	1	1
Female	**1.52 (1.18, 1.96)**	1.27 (0.97, 1.67)	1.43 (0.98, 2.08)	1.20 (0.78, 1.85)
Anxiety/depression				
Not anxious or depressed	1	1	1	1
Anxious or depressed	**2.92 (2.28, 3.74)**	**2.48 (1.84, 3.35)**	**4.69 (3.17, 6.94)**	**3.14 (2.09, 4.71)**
Pain sites:				
None	1	1	1	1
1-3	**3.01 (2.01, 4.54)**	**2.87 (1.90, 4.33)**	1.77 (0.81, 3.88)	1.53 (0.71, 3.31)
4+	**4.43 (3.03, 6.48)**	**3.53 (2.37, 5.26)**	**5.89 (3.20, 10.85)**	**3.91 (1.99, 7.68)**
Between person variance	-	6.07	-	128.51

For peer review only

Supplementary Table 4: Results from multiple imputation analysis of associations with monthly SoB response

Multiple Imputation	Monthly SOB			
Baseline characteristics	1-3 weeks *vs.* none		4 weeks *vs.* none	
	Unadjusted RRR (95% CI)	Adjusted[a] RRR (95% CI)	Unadjusted RRR (95% CI)	Adjusted[a] RRR (95% CI)
Age				
<60	1	1	1	1
60-69	1.37 (0.92, 2.05)	1.36 (0.90, 2.06)	1.05 (0.65, 1.69)	1.10 (0.66, 1.82)
70-79	1.79 (1.22, 2.63)	1.91 (1.29, 2.83)	1.34 (0.85, 2.12)	1.65 (1.01, 2.69)
80+	1.92 (1.25, 2.96)	2.33 (1.50, 3.61)	1.36 (0.80, 2.30)	2.12 (1.21, 3.70)
BMI				
Normal	1	1	1	1
Overweight	1.24 (0.95, 1.62)	1.32 (1.00, 1.74)	1.02 (0.74, 1.41)	1.09 (0.77, 1.54)
Obese	1.51 (1.11, 2.06)	1.56 (1.11, 2.20)	2.61 (1.84, 3.70)	2.60 (1.77, 3.82)
Gender				
Male	1	1	**1**	1
Female	**1.75 (1.38, 2.22)**	**1.40 (1.09, 1.80)**	**2.15 (1.61, 2.88)**	1.59 (1.16, 2.18)
Anxiety/depression				
Not anxious or depressed	1	1	1	1
Anxious or depressed	**2.93 (2.25, 3.82)**	**2.49 (1.88, 3.31)**	**5.16 (3.84, 6.94)**	**4.05 (2.89, 5.68)**
Pain sites:				
None	1	1	1	1
1-3	**2.17 (1.52, 3.10)**	**1.99 (1.36, 2.90)**	3.77 (2.24, 6.35)	3.09 (1.78, 5.36)
4+	**3.38 (2.35, 4.86)**	**2.61 (1.75, 3.90)**	6.44 (3.91, 10.63)	**3.93 (2.24, 6.89)**
Between person variance	-	5.08	-	14.80

For peer review only

Supplementary Table 5: Summary of the clusters (8-cluster model) including patients responding to at least 2 monthly questionnaires

Description	Cluster 1 No symptoms	Cluster 2 Infrequent SOB	Cluster 3 Occasional pain	Cluster 4 Occasional SOB	Cluster 5 Occasional pain & SOB	Cluster 6 Frequent pain & SOB	Cluster 7 Persistent SOB	Cluster 8 Persistent pain & SOB
Observed *n* (%)	189 (24)	106 (14)	20 (3)	89 (11)	161 (21)	96 (12)	66 (8)	50 (6)
Range of monthly probabilities of extent of chest pain / SOB								
Chest pain	0.95-1.00	0.85-0.90	0.14-0.24	0.85-0.91	0.22-0.25	0.06-0.11	0.83-0.93	<0.01
None								
Up to 3 weeks	<0.01-0.05	0.10-0.14	0.66-0.68	0.09-0.14	0.66-0.68	0.58-0.68	0.07-0.16	0.04-0.11
4 weeks	<0.01	<0.01	0.10-0.20	<0.01	0.09-0.11	0.21-0.36	<0.01	0.89-0.96
SOB	0.96-0.99	0.51-0.65	0.76-0.93	0.13-0.18	0.14-0.15	0.01-0.02	<0.01	<0.01
None								
Up to 3 weeks	0.01-0.04	0.33-0.47	0.07-0.23	0.73-0.74	0.73-0.74	0.33-0.43	0.11-0.16	0.03-0.11
4 weeks	<0.01	0.01-0.02	<0.01	0.10-0.13	0.11-0.13	0.55-0.66	0.83-0.87	0.88-0.96

SOB: shortness of breath

For peer review only

Supplementary Table 6: Comparison between clusters on baseline socio-demographic and general health measures using multiple imputed data (m=50)

Description	Cluster 1 No symptoms	Cluster 2 Infrequent SOB	Cluster 3 Occasional pain	Cluster 4 Occasional SOB	Cluster 5 Occasional pain & SOB	Cluster 6 Frequent pain & SOB	Cluster 7 Persistent SOB	Cluster 8 Persistent pain & SOB
Observed *%*	22	14	3	12	18	14	9	8
Female *n* (%)	24	40	30	42	38	43	50	41
Age <60	13	7	21	8	10	6	5	20
60-69	33	26	29	23	29	21	25	32
70-79	37	39	37	40	39	39	41	35
80+	17	28	14	30	22	34	30	13
BMI group								
Normal weight	31	28	51	22	29	29	20	23
Overweight	50	53	34	47	46	40	30	32
Obese	19	18	15	31	25	31	49	44
PCS mean	45.5 (43.9, 47.1)	37.7 (35.6, 39.7)	38.9 (34.8, 42.9)	36.7 (34.6, 38.7)	35.7 (34.0, 37.3)	27.6 (25.9, 29.3)	29.8 (27.4, 32.2)	25.0 (22.8, 27.2)
Pain sites								
None	26	17	13	8	7	5	12	3
1-3 sites	49	44	44	37	35	27	45	20
4 plus sites	25	39	43	55	58	68	43	76
Anxious or depressed	16	35	38	49	52	77	50	73

SOB: shortness of breath. 50 imputations: model included baseline PCS score, anxiety, depression chest pain, SOB, cluster, age, gender, heart failure, number of pain sites

For peer review only

STROBE Statement—checklist of items that should be included in reports of observational studies

	Item No	Recommendation
**Title and abstract**	1	(*a*) Indicate the study's design with a commonly used term in the title or the abstract **[within the title page 1]**
		(*b*) Provide in the abstract an informative and balanced summary of what was done and what was found **[within the abstract under results section]**
**Introduction**		
Background/rationale	2	Explain the scientific background and rationale for the investigation being reported **[page 3]**
Objectives	3	State specific objectives, including any prespecified hypotheses **[pages 3-4]**
**Methods**		
Study design	4	Present key elements of study design early in the paper **[p 4]**
Setting	5	Describe the setting, locations, and relevant dates, including periods of recruitment, exposure, follow-up, and data collection **[page 4]**
Participants	6	(*a*) *Cohort study*—Give the eligibility criteria, and the sources and methods of selection of participants. Describe methods of follow-up **[pages 4-5]**<br>*Case-control study*—Give the eligibility criteria, and the sources and methods of case ascertainment and control selection. Give the rationale for the choice of cases and controls<br>*Cross-sectional study*—Give the eligibility criteria, and the sources and methods of selection of participants
		(*b*) *Cohort study*—For matched studies, give matching criteria and number of exposed and unexposed<br>*Case-control study*—For matched studies, give matching criteria and the number of controls per case
Variables	7	Clearly define all outcomes, exposures, predictors, potential confounders, and effect modifiers. Give diagnostic criteria, if applicable **[pages 5-6]**
Data sources/ measurement	8*	For each variable of interest, give sources of data and details of methods of assessment (measurement). Describe comparability of assessment methods if there is more than one group **[pages 5-6]**
Bias	9	Describe any efforts to address potential sources of bias **[N/A]**
Study size	10	Explain how the study size was arrived at **[pages 8-9]**
Quantitative variables	11	Explain how quantitative variables were handled in the analyses. If applicable, describe which groupings were chosen and why **[pages 5-6]**
Statistical methods	12	(*a*) Describe all statistical methods, including those used to control for confounding **[pages 6-8]**
		(*b*) Describe any methods used to examine subgroups and interactions **[pages 6-8]**
		(*c*) Explain how missing data were addressed **[page 8]**
		(*d*) *Cohort study*—If applicable, explain how loss to follow-up was addressed **[page 8]**<br>*Case-control study*—If applicable, explain how matching of cases and controls was addressed<br>*Cross-sectional study*—If applicable, describe analytical methods taking account of sampling strategy
		(*e*) Describe any sensitivity analyses **[page 8]**

For peer review only - http://bmjopen.bmj.com/site/about/guidelines.xhtml

Continued on next page

For peer review only

For peer review only - http://bmjopen.bmj.com/site/about/guidelines.xhtml

**Results**		
Participants	13*	(a) Report numbers of individuals at each stage of study—eg numbers potentially eligible, examined for eligibility, confirmed eligible, included in the study, completing follow-up, and analysed **[pages 8-9]**
		(b) Give reasons for non-participation at each stage **[N/A]**
		(c) Consider use of a flow diagram **[N/A]**
Descriptive data	14*	(a) Give characteristics of study participants (eg demographic, clinical, social) and information on exposures and potential confounders **[pages 8-9]**
		(b) Indicate number of participants with missing data for each variable of interest **[N/A]**
		(c) *Cohort study*—Summarise follow-up time (eg, average and total amount) **[page 8]**
Outcome data	15*	*Cohort study*—Report numbers of outcome events or summary measures over time **[pages 8-9]**
		Case-control study—Report numbers in each exposure category, or summary measures of exposure
		Cross-sectional study—Report numbers of outcome events or summary measures
Main results	16	(*a*) Give unadjusted estimates and, if applicable, confounder-adjusted estimates and their precision (eg, 95% confidence interval). Make clear which confounders were adjusted for and why they were included **[pages 9-11]**
		(*b*) Report category boundaries when continuous variables were categorized **[pages 9-11]**
		(*c*) If relevant, consider translating estimates of relative risk into absolute risk for a meaningful time period **[N/A]**
Other analyses	17	Report other analyses done—eg analyses of subgroups and interactions, and sensitivity analyses **[pages 9-12]**
**Discussion**		
Key results	18	Summarise key results with reference to study objectives **[pages 11-12]**
Limitations	19	Discuss limitations of the study, taking into account sources of potential bias or imprecision. Discuss both direction and magnitude of any potential bias **[pages 12-13]**
Interpretation	20	Give a cautious overall interpretation of results considering objectives, limitations, multiplicity of analyses, results from similar studies, and other relevant evidence **[page 13]**
Generalisability	21	Discuss the generalisability (external validity) of the study results **[pages 12-13]**
**Other information**		
Funding	22	Give the source of funding and the role of the funders for the present study and, if applicable, for the original study on which the present article is based **[page 14]**

*Give information separately for cases and controls in case-control studies and, if applicable, for exposed and unexposed groups in cohort and cross-sectional studies.

Note: An Explanation and Elaboration article discusses each checklist item and gives methodological background and published examples of transparent reporting. The STROBE checklist is best used in conjunction with this article (freely available on the Web sites of PLoS Medicine at http://www.plosmedicine.org/, Annals of Internal Medicine at http://www.annals.org/, and Epidemiology at http://www.epidem.com/). Information on the STROBE Initiative is available at www.strobe-statement.org.

For peer review only

BMJ Open

Chest pain and shortness of breath in cardiovascular disease: a prospective cohort study in UK Primary Care

Journal:	*BMJ Open*
Manuscript ID	bmjopen-2017-015857.R1
Article Type:	Research
Date Submitted by the Author:	08-Mar-2017
Complete List of Authors:	Barnett, Lauren; Keele University, Arthritis Research UK Primary Care Centre<br>Prior, James A.; Keele Univ<br>Kadam, Umesh; Keele University, Health Services Research Unit<br>Jordan, Kelvin; Keele University, Arthritis Research UK Primary Care Centre
**Primary Subject Heading**:	Cardiovascular medicine
Secondary Subject Heading:	Epidemiology
Keywords:	cardiovascular disease, chest pain, Ischaemic heart disease < CARDIOLOGY, Heart failure < CARDIOLOGY, shortness of breath

SCHOLARONE™
Manuscripts

For peer review only

Title: Chest pain and shortness of breath in cardiovascular disease: a prospective cohort study in UK Primary Care

Barnett LA, Prior JA, Kadam UT, Jordan KP

Authors

Lauren A. Barnett, NIHR Research Methods Fellow, MSc[1]

*James A. Prior, Research Associate, PhD[1]

Umesh T. Kadam, Professor in Clinical Epidemiology & Health Services Research, PhD[2]

Kelvin P. Jordan, Professor of Biostatistics, PhD[1]

Affiliation

[1]Research Institute for Primary Care and Health Sciences, Keele University, UK

[2]Keele Cardiovascular Research Group, Institute for Applied Clinical Sciences, Keele University, UK

***Corresponding author:**

James A. Prior

Research Institute for Primary Care and Health Sciences,

Keele University, Staffordshire, United Kingdom,

ST5 5BG

E-mail: j.a.prior@keele.ac.uk

Tel: +44 (0) 1782 734847

For peer review only - http://bmjopen.bmj.com/site/about/guidelines.xhtml

Abstract

Objective: To determine characteristics associated with monthly chest pain and shortness of breath (SoB) during activity in cardiovascular disease (CVD), and trajectories of these symptoms over 10 months.

Study Design and Setting: Baseline questionnaire was sent to patients aged ≥40 years from 10 UK general practices. Responders were sent monthly questionnaires for 10 months. For patients with CVD (ischaemic heart disease; heart failure), the association of socio-demographic characteristics, pain elsewhere, and anxiety & depression with monthly reports of chest pain and SoB during activity were determined using multilevel multinomial logistic regression. Common symptom trajectories were determined using dual trajectory latent class growth analysis.

Results: 661 patients with CVD completed at least five monthly questionnaires. Multiple other pain sites (relative risk ratio 4.03; 95% CI 1.64, 9.91) and anxiety or depression (3.31; 1.89, 5.79) were associated with reporting weekly chest pain. Anxiety or depression (4.10; 2.72, 6.17), obesity (2.53; 1.49, 4.30), older age (80+: 2.51; 1.19, 5.26), increasing number of pain sites (4+: 4.64; 2.35, 9.18), and female gender (1.81; 1.20, 2.75) were associated with reporting weekly SoB. Eight symptom trajectories were identified with SOB symptoms more common than chest pain.

Conclusions: Potentially modifiable characteristics are associated with the experience of chest pain and SoB. Identified symptom trajectories may facilitate tailored care to improve outcomes in patients with CVD.

For peer review only - http://bmjopen.bmj.com/site/about/guidelines.xhtml

Strengths & Limitations of this study: This was a large study of primary care patients with diagnosed cardiovascular (CVD) disease. It is the first study to examine the course of CVD symptoms based on monthly self-reported data. There was attrition, with many patients not completing sufficient monthly questionnaires to be included in the analysis. Due to the nature of the questionnaire data we were unable to identify the number or the duration of the monthly symptom episodes.

For peer review only

For peer review only - http://bmjopen.bmj.com/site/about/guidelines.xhtml

1. Introduction

Chest pain and shortness of breath (SoB) are common symptoms of cardiovascular disease (CVD) seen in UK primary care. The incidence of chest pain ranges between 13-20 per 1,000 person years (1-3), with chest pain consultation being a risk factor for future cardiovascular morbidity and mortality (2,4). Shortness of breath is a frequent symptom of heart failure (HF) and angina (5,6). Patients with CVD may experience and consult for chest pain and SoB separately, whilst many patients will experience both together (7).

Though directly associated with CVD, chest pain and SoB may also be experienced due to non-cardiac reasons. In a US sample of 10,881 participants with no prevalent CVD or pulmonary conditions, 22% reported SoB (8) and SoB and chest pain has been shown to be associated with other patient characteristics such as age, gender and depression (9). Understanding potentially modifiable patient characteristics associated with the symptoms of chest pain and SoB (whether related to CVD pathology or not) in patients diagnosed with CVD may present additional opportunities to direct management to reduce the risk of disease progression and prolonged symptom experience, and improve outcomes in patients with CVD.

Symptoms of chest pain and SoB are not typically isolated events. Assessing how patients' symptoms co-occur and change over time may help identify common patterns (trajectories) of these symptoms which may be related to long-term outcomes. However, how the experience of chest pain and SoB in patients with CVD varies over time remains unclear. Other trajectories of symptoms have previously been determined to aid understanding of the course of morbidity, for example in back pain (10). Understanding the different trajectories of patient symptoms can be beneficial when trying to optimise health care resources, for example through stratified care (11). Such

For peer review only - http://bmjopen.bmj.com/site/about/guidelines.xhtml

methods may allow better targeting of interventions, potentially earlier or to those with greater need.

The objectives of our study, in a primary care population with CVD, were; i) to determine the association of patient characteristics with the monthly experience of chest pain or SoB during activity and ii) to determine common trajectories of chest pain and SoB symptoms over time.

2. Methods

2.1. Study design

This analysis uses data from the Comorbidity Cohort (2C) study. A prospective cohort study, this was designed to investigate the interaction between CVD and osteoarthritis (OA) comorbidity over 12 months. Full details of the 2C study are reported elsewhere (12-14). In brief, the 2C study sampled patients with recorded cardiovascular morbidity and/or OA, aged 40 years or older, plus a reference group without CVD or OA, from the electronic health records of 10 UK general practices. All patients fulfilling the study inclusion criteria were mailed a baseline questionnaire, which included measures of general health, as well as CVD and OA specific measures. Those who completed the baseline questionnaire and consented to further follow-up were mailed shorter monthly health questionnaires for the following 10 months and a larger questionnaire at 12 months.

2.2. Study Population

This analysis focuses only on those patients with recorded CVD (with or without comorbid OA) of which there were 2,858 sent a baseline questionnaire. These patients were identified through a primary care record of ischaemic heart disease (IHD) or heart

For peer review only - http://bmjopen.bmj.com/site/about/guidelines.xhtml

failure (HF) using Read Codes (commonly used to record morbidity in UK primary care) within a 3-year period prior to the baseline questionnaire, starting from November 2006. IHD was identified by Read codes starting G3 (“Ischaemic heart disease”), and HF was identified by Read codes starting G58 (“Heart failure”) and codes related to the New York Heart Association (NYHA) classification (codes 662f-i).

2.3. Outcome measures

The primary outcomes were monthly self-reported measures of two CVD-related symptoms: i) chest pain during activity and ii) SoB during activity. A single item question was used for each symptom, both based on existing, valid outcome measures (Seattle Angina Questionnaire (SAQ) (15) and the Kansas City Cardiomyopathy Questionnaire (KCCQ) (16)). Patients were asked “How often (if at all) have you had chest pain during activity over the past 4 weeks?”. The same question structure was used for SoB. Response options for these questions were “Not at all”, “For 1 week or less”, “For 2 weeks”, “For 3 weeks”, and “For 4 weeks. For analysis, these were categorised into: “not at all”, “for 1-3 weeks”, and “for 4 weeks”, representing no, episodic, and persistent monthly symptoms.

2.4. Covariates

Covariates measured in the baseline questionnaire were age, gender, body mass index (BMI, based on self-reported height and weight), general physical health, pain elsewhere, and anxiety and depression. BMI was categorised as: normal weight (<25), overweight (25-30) or obese (30+). General physical health was measured using the 12-item Short Form Health Survey (SF-12) (17). From this, the Physical Component Summary (PCS) score was derived; this is normalized to the US general population with

For peer review only - http://bmjopen.bmj.com/site/about/guidelines.xhtml

scores below 50 reflecting worse physical health than for the general population. Anxiety and depression were measured using the Hospital Anxiety and Depression scale with the possible score ranging from 0 (best) to 21 (worst) for anxiety and for depression (18). Given the strong association between anxiety and depression, we combined the two scales and defined those with borderline or probable anxiety or depression (based on standard cut-offs) as anxious or depressed. We determined the extent of pain elsewhere at baseline as a count of 10 self-reported areas of pain (neck, shoulder, elbow, hand, back, hip, knee, foot, abdominal and headache). This was categorised into none, 1-3, and 4 or more sites. Lacey et al. (2015) suggested the number of self-reported pain sites is more important than the pain location (19). Baseline chest pain and SoB were measured using yes/no responses to items enquiring about any chest pain or tightness in the past 4 weeks, and any shortness of breath in the past 4 weeks.

2.5. Statistical Analyses

The statistical analysis was performed in two phases. The first phase examined associations between baseline sample characteristics and monthly chest pain and SoB experienced during activity. The second phase clustered patients into groups based on the most common patterns (trajectories) of chest pain and SoB experienced over the 10-month follow-up time-period. Responders who had completed chest pain or SoB items on at least 5 of the 10 monthly questionnaires were included in the analyses.

2.5.1. Phase one – Associations between baseline characteristics, chest pain and SoB

A multilevel multinomial logistic regression model (monthly measurements nested within respondents) was used to determine associations between the baseline sample

For peer review only - http://bmjopen.bmj.com/site/about/guidelines.xhtml

characteristics (age, gender, BMI, general physical health, pain elsewhere, anxiety and depression) and monthly chest pain during activity (1-3 weeks vs. none, and 4+ weeks vs. none). Associations were initially unadjusted, followed by adjustment for covariates. PCS score was not included in the multivariable model due to strong association with age and pain elsewhere. Interactions between time and covariates that were found to be statistically significant were incorporated into the model to assess whether their effects changed over the time period. The same analysis was then repeated for SoB.

Sensitivity analyses were performed using respondents who completed the chest pain and SoB items on at least 2 monthly questionnaires and by using multiple imputation (with 50 imputations) to impute the missing monthly questionnaire answers and any missing covariate data.

2.5.2 Phase two – Chest pain and SoB cluster trajectories

Dual trajectory Latent Class Growth Analysis (LCGA) was carried out to group (cluster) respondents into the most common trajectories of self-reported chest pain and SoB over the 10 months using the repeated monthly measures of the two symptoms (20). Each cluster, therefore, represents a common trajectory of both chest pain and SoB over the 10 months. A fundamental assumption behind dual trajectory LCGA is that the two symptoms, in this case, chest pain and SoB, are correlated. The time-order of the measurements were taken into account when deriving the trajectories within a cluster by using quadratic growth curves.

There is no definitive method of deciding on the most appropriate number of clusters using statistical goodness of fit measures. We initially used Akaike's information criterion (AIC), Bayesian information criterion (BIC), and the adjusted BIC and for each of these, the model with the lowest goodness of fit value indicates the optimal number

For peer review only - http://bmjopen.bmj.com/site/about/guidelines.xhtml

of clusters. The final decision on the optimal number of clusters was determined by a combination of statistical information, the size and distinctiveness of the clusters, and how well the profile (monthly responses of chest pain and SoB) of respondents allocated to each cluster matched that for the cluster as a whole. Respondents were allocated to the cluster for which they had the highest posterior probability of belonging. The mean of these posterior probabilities for respondents assigned to each cluster (i.e. cluster-specific average posterior probabilities (AvePP)) was determined. An AvePP greater than 0.7 suggests clear classification of participants into that cluster (21). Cluster-specific probabilities of reporting of chest pain and SoB for each month allow profiles of the trajectory of CVD symptoms to be developed for each cluster. The clusters were then descriptively compared on the baseline covariates described in Section 2.4.

As a sensitivity analysis, LCGA was repeated using participants completing the chest pain and SoB items on at least 2 monthly questionnaires. A further sensitivity analysis used multiple imputation to impute clusters for all baseline CVD respondents, with 50 imputations, and using baseline age, gender, BMI, anxiety, depression, PCS score, number of pain sites elsewhere and baseline chest pain, SoB and heart failure diagnosis in the imputation. All analysis was done using Stata 14, Mplus version 7.3 and MLwiN 2.35.

3. Results

3.1. Patient demographics

2,858 CVD patients were mailed the baseline questionnaire, of which 1,696 (59%) responded. 661 (39%) baseline responders answered the chest pain and SoB items on between 5 and 10 monthly questionnaires and were included in the main analyses

For peer review only - http://bmjopen.bmj.com/site/about/guidelines.xhtml

reported here. Compared to all other baseline responders, those in the analysis were younger (mean difference 2.3 years), had a higher proportion of males (68% vs 62%), and had a slightly lower prevalence of chest pain (42% vs 44%) and SoB (54% vs 58%) at baseline (**Table 1**).

During the 10-month follow-up period, prevalence of chest pain during activity in all 4 weeks ranged from 11% to 13% by month, and that of chest pain for 1 to 3 weeks from 23% to 28%. Monthly prevalence of SoB during activity in all 4 weeks ranged from 21% to 26%, and SoB for 1-3 weeks from 34% to 37%.

3.2.1 Phase one - Associations between baseline characteristics and chest pain

Unadjusted analysis showed increasing number of pain sites elsewhere, being anxious or depressed, and having a worse PCS score were associated with an increase in risk of reporting chest pain in all 4 weeks in a month.

After adjustment for all covariates, an increasing number of pain sites (relative risk ratio (RRR) for 4+ vs. none: 4.03, 95% CI: 1.64, 9.91) and being anxious or depressed (RRR: 3.31, 95% CI: 1.89, 5.79) were associated with reporting chest pain in all 4 weeks in the previous month (**Table 2**). The interactions of anxiety or depression, and number of pain sites at baseline with time were not statistically significant.

3.2.2 Phase one - Associations between baseline characteristics and SoB

Unadjusted analysis showed more pain sites elsewhere, being anxious or depressed, obesity, female gender, and having a worse PCS score were associated with an increase in risk of reporting SOB during activity in all 4 weeks in the last month.

After adjustment for all covariates, increasing number of pain sites (RRR for 4+ vs. none: 4.64, 95% CI: 2.35, 9.18), being anxious or depressed (RRR: 4.10, 95% CI: 2.72,

For peer review only - http://bmjopen.bmj.com/site/about/guidelines.xhtml

6.17), and obesity (RRR: 2.53, 95% CI: 1.49, 4.30) were associated with increased risk of reporting SOB in each of the previous 4 weeks, as were female gender and being over 80 years of age. There were no statistically significant interactions with time (**Table 3**).

3.2.3 Phase one - Sensitivity analyses

Sensitivity analyses including those responding on at least 2 monthly questionnaires (*n*=777) and using multiple imputation did not change the findings (**Supplementary Tables 1-4**).

For peer review only

3.3 Phase two – Trajectories of chest pain and SOB

The goodness of fit measures from the LCGA did not clearly suggest an optimal number of clusters of trajectories of chest pain and SOB, although their values levelled off from the model with eight clusters (**Table 4**). The six-cluster model had a lowest observed cluster size of 7% (*n*=47) of participants, compared to 4% (24 participants) for the seven-cluster model, and 3% (20 participants) for the model with eight clusters. Based on the goodness of fit measures, the size of clusters and their interpretation, the eight-cluster model was preferred (**Table 5**).

Patients allocated to the largest cluster (*n*=166, 25%) generally reported no pain or SoB symptoms (with monthly probability 0.95 and above) in all the monthly questionnaires. Across clusters, in those reporting symptoms, SoB was more common than chest pain. One cluster of patients reported infrequent breathlessness symptoms (reporting SoB for up to 3 weeks on a median of 3 of the 10 monthly questionnaires: *n*=93, 14%), another cluster of patients reported occasional breathlessness symptoms (reporting SoB for up to 3 weeks in a median of 8 out of the 10 months: *n*=83, 13%) and a third cluster of patients reported persistent breathlessness symptoms (reporting SoB in all of the last 4

For peer review only - http://bmjopen.bmj.com/site/about/guidelines.xhtml

weeks in all monthly questionnaires: *n*=52, 8%), but people in these three clusters rarely reported chest pain. The smallest cluster reported occasional chest pain, but rarely reported SoB (*n*=20, 3%). Two clusters reported occasional (*n*=125, 19%) or frequent (no months without symptoms, *n*= 75, 11%) pain alongside breathlessness. The most severe group of patients reported persistent SoB and pain over the 10 months, i.e. reported SoB and pain for all four weeks in all monthly questionnaires (*n*=47, 7%). Patients in the clusters with both pain and SoB that was frequent or persistent had poorer baseline comorbid symptoms (anxiety and depression, and pain elsewhere) and worse physical health in general (**Table 6**). Levels of obesity were higher in those with persistent SoB (with or without pain). The most severe group were younger than the other symptomatic groups.

Sensitivity analysis, including respondents with at least 2 monthly responses, showed a similar distribution of people across clusters (**Supplementary Table 5**). Multiple imputation of cluster membership for all baseline respondents reduced the percentage in the no symptoms cluster (25% to 22%) and increased the percentage in the frequent pain and SoB cluster (11% to 14%) (**Supplementary Table 6**).

4 Discussion

4.1 Summary

Using a prospective cohort study of patients with cardiovascular disease, we found that potentially modifiable characteristics (weight, psychological health, and number of painful body sites) influence the experience of both occasional and persistent chest pain and shortness of breath during activity, and that eight distinct symptom trajectories are apparent, with breathlessness symptoms more common than chest pain.

For peer review only

Three of our identified clusters consisted of patients with stable conditions. The most common cluster (containing a quarter of our sample) reported no symptoms every month. By contrast, one cluster reported persistent chest pain and SoB every month, and another reported persistent SoB without chest pain every month. Symptoms of patients in the remaining clusters fluctuated more. We did not observe a cluster who improved or worsened over the 10 months, which may reflect the prevalent rather than incident nature of the cohort.

Though chest pain and shortness of breath may be considered a distinctive symptom of acute CVD conditions, such as myocardial infarction, a quarter of our prevalent CVD sample did not experience these symptoms at all, similar to a study of community recruited HF patients from the UK (22). Chest pain and SoB commonly co-occurred, but SoB was more common and was reported without chest pain in three of our clusters (containing over a third of our cohort). We found a strong association between poor psychological health and both chest pain and shortness of breath. Our findings are supported by previous cross-sectional studies which showed a correlation between shortness of breath and depression both in US and Chinese secondary care heart failure samples (23,24). Approximately two-thirds of patients with CVD in the most severe symptom clusters (frequent or persistent chest pain and SoB) had anxiety or depression. These results are supported by longitudinal research which reported poorer treatment outcomes in angina patients with chest pain and depression, compared to those without depression (25) and that in angina patients with persistent symptoms, long-term anxiety and depression were more likely (26). Our research shows that the psychological health of CVD patients, especially those with frequent chest pain and SoB is poor. However, at present our findings only indicate the association between chest pain, SoB and poor mental health. It remains unclear whether

For peer review only - http://bmjopen.bmj.com/site/about/guidelines.xhtml

interventions aimed at anxiety or depression would improve these CVD symptoms, though this does present a potential avenue for future research.

CVD patients who were obese at baseline were more likely to experience SoB compared to those of normal weight. This in line with other research, and also demonstrates potential to improve this common CVD symptom. Bernhardt and Babb (2014) found that moderate weight loss in women who were obese at baseline was effective at reducing breathlessness during exertion (27).

We have shown that chest pain and SoB are associated with anxiety and depression and pain elsewhere, and addressing these characteristics may benefit both symptoms. Clark et al reported that improving (or diminishing) SoB after two years depended on whether pain resolved or diminished respectively (7). As a third of our sample reported both chest pain and SoB, these present a considerable proportion of patients with CVD where future research may look to develop interventions towards a singular problem (for example, anxiety) which may also benefit the two symptoms.

Our clusters provide distinct patterns of symptom experience over time within a CVD population. Three quarters of CVD patients experienced either shortness of breath or chest pain; however, approximately a third of our sample (35%) reported SoB only, either infrequently (14%), occasionally (13%) or persistently (8%). These may represent a cluster of patients at greater risk of poor outcomes. In a large sample of US participants with coronary artery disease at baseline, the mortality rates were twice as high in patients with SoB compared to those with other symptoms and four times greater than in those with no symptoms at presentation (28). Symptoms such a chest pain and SoB may act as prognostic indicators for outcomes resulting in (8), or from CVD (28) and therefore our findings present a range of likely trajectories to which future research may tailor interventions.

For peer review only - http://bmjopen.bmj.com/site/about/guidelines.xhtml

4.1 Strengths & limitations

The strength of our study lies in its prospective cohort design which has used a large CVD sample from primary care to gain an insight into the monthly experience of chest pain and SoB symptom in this sample. This study was conducted within a UK primary care setting using monthly surveys from patients with a diagnosis of CVD, identified through electronic health records from 10 general practices. Since these patients were identified from general practices across North Staffordshire, Stoke-on-Trent and Cheshire they represent a sample generalisable to the UK primary care population (29). This is the first research to examine monthly trajectories of these symptoms in CVD patients.

There are several limitations to our work. Firstly, due to the prospective nature of our research there was attrition during the follow-up period. Those in the monthly analysis were slightly younger and more likely to be male than all those responding at baseline. Related to attrition, our use of multiple imputation may be limited as we are unable to determine if missing values are truly missing at random. In the monthly questionnaire, our definitions of chest pain and SoB were based on a single question for each. Though using a short monthly questionnaire improved the month-on-month response rates, such a simple definition may limit the accuracy of patient responses. The questions related to chest pain and SoB that occurred during activity; however, it is possible that the symptoms may be non-cardiovascular in origin. We also did not identify the number or duration of symptom episodes other than in how many weeks they had occurred in the last month. However, no matter the specific cause of the chest pain or shortness of breath, patients with CVD are experiencing these symptoms and therefore these need to be addressed.

For peer review only - http://bmjopen.bmj.com/site/about/guidelines.xhtml

4.2 Conclusions

Several modifiable patient characteristics (weight, psychological health, and number of other painful body sites) are associated with the experience of both occasional and weekly chest pain and shortness of breath during activity. Future interventions to improve CVD symptom experience may wish to target these covariates. By identifying trajectories of symptoms over time, care may be better tailored to improve outcomes in those patients with CVD at greatest risk of worse symptoms and disease progression.

For peer review only

For peer review only - http://bmjopen.bmj.com/site/about/guidelines.xhtml

Acknowledgements: We are grateful for the participation of general practice teams and their patients in supporting the 2C study. Acknowledgements are given to the Keele survey, network, administration and management teams who supported the study.

Contribution statement: Guarantor of overall study integrity: KPJ. Study concept & design: KPJ, JAP & UTK. Data collection and interpretation: LAB, KPJ, JAP & UTK. Statistical analysis: LAB & KPJ. Manuscript preparation: LAB, KPJ, JAP & UTK. Final approval of manuscript: LAB, KPJ, JAP & UTK.

Funding: LAB is funded by an NIHR Research Methods Fellowship and UTK was funded by a NIHR Post-Doctoral Fellowship (grant no. PAS/PDA/03/07/035).
The study sponsors had no role in study design; in the collection, analysis, and interpretation of data; in the writing of the report; and in the decision to submit the paper for publication. The views expressed are those of the authors and not necessarily those of the NHS, the NIHR or the Department of Health.

Competing interests: The authors have no conflicts of interest

Ethics approval: Ethics approval for the 2C study was granted by Cheshire Research Ethics Committee (reference number 09/H1017/40).

Data sharing: No additional data

For peer review only - http://bmjopen.bmj.com/site/about/guidelines.xhtml

References

(1) Walters K, Rait G, Hardoon S, Kalaitzaki E, Petersen I, Nazareth I. Socio-demographic variation in chest pain incidence and subsequent coronary heart disease in primary care in the United Kingdom. European Journal of Preventive Cardiology 2014 May 01;21(5):566-575.

(2) Ruigómez A, Rodríguez LAG, Wallander M, Johansson S, Jones R. Chest pain in general practice: incidence, comorbidity and mortality. Family Practice 2006 April 01;23(2):167-174.

(3) Ruigomez A, Masso-Gonzalez EL, Johansson S, Wallander MA, Garcia-Rodriguez LA. Chest pain without established ischaemic heart disease in primary care patients: associated comorbidities and mortality. Br J Gen Pract 2009 Mar;59(560):e78-86.

(4) Robson J, Ayerbe L, Mathur R, Addo J, Wragg A. Clinical value of chest pain presentation and prodromes on the assessment of cardiovascular disease: a cohort study. BMJ Open 2015 Apr 15;5(4):e007251-2014-007251.

(5) van Riet EES, Hoes AW, Limburg A, Landman MAJ, van der Hoeven H, Rutten FH. Prevalence of unrecognized heart failure in older persons with shortness of breath on exertion. European Journal of Heart Failure 2014;16(7):772-777.

(6) Task Force Members, Montalescot G, Sechtem U, Achenbach S, Andreotti F, Arden C, et al. 2013 ESC guidelines on the management of stable coronary artery disease: the Task Force on the management of stable coronary artery disease of the European Society of Cardiology. Eur Heart J 2013 Oct;34(38):2949-3003.

(7) Clark N, Fan VS, Slatore CG, Locke E, Whitson HE, Nici L, et al. Dyspnea and pain frequently co-occur among Medicare managed care recipients. Ann Am Thorac Soc 2014 Jul;11(6):890-897.

(8) Santos M, Kitzman DW, Matsushita K, Loehr L, Sueta CA, Shah AM. Prognostic Importance of Dyspnea for Cardiovascular Outcomes and Mortality in Persons without Prevalent Cardiopulmonary Disease: The Atherosclerosis Risk in Communities Study. PLoS One 2016 Oct 25;11(10):e0165111.

(9) Lenfant C. Chest pain of cardiac and noncardiac origin. Metab Clin Exp 2010 10;59, Supplement 1:S41-S46.

(10) Dunn KM, Campbell P, Jordan KP. Long-term trajectories of back pain: cohort study with 7-year follow-up. BMJ Open 2013 December 01;3(12).

(11) Hill JC, Whitehurst DG, Lewis M, Bryan S, Dunn KM, Foster NE, et al. Comparison of stratified primary care management for low back pain with current best practice (STarT Back): a randomised controlled trial. Lancet 2011 Oct 29;378(9802):1560-1571.

For peer review only

For peer review only - http://bmjopen.bmj.com/site/about/guidelines.xhtml

(12) Prior JA, Rushton CA, Jordan KP, Kadam UT. Comorbidity Cohort (2C) study: Cardiovascular disease severity and comorbid osteoarthritis in primary care. BMC Health Serv Res 2012 Sep 3;12(1):295.

(13) Prior JA, Jordan KP, Kadam UT. Associations between cardiovascular disease severity, osteoarthritis co-morbidity and physical health: a population-based study. Rheumatology 2014 May 11.

(14) Rushton CA, Kadam UT. Impact of non-cardiovascular disease comorbidity on cardiovascular disease symptom severity: A population-based study. Int J Cardiol 2014 7/15;175(1):154-161.

(15) Dougherty CM, Dewhurst T, Nichol WP, Spertus J. Comparison of three quality of life instruments in stable angina pectoris: Seattle Angina Questionnaire, Short Form Health Survey (SF-36), and Quality of Life Index-Cardiac Version III. J Clin Epidemiol 1998 Jul;51(7):569-575.

(16) Spertus J, Peterson E, Conard MW, Heidenreich PA, Krumholz HM, Jones P, et al. Monitoring clinical changes in patients with heart failure: A comparison of methods. American Heart Journal 2005 10;150(4):707-715.

(17) Ware J,Jr, Kosinski M, Keller SD. A 12-Item Short-Form Health Survey: construction of scales and preliminary tests of reliability and validity. Med Care 1996 Mar;34(3):220-233.

(18) Zigmond AS, Snaith RP. The hospital anxiety and depression scale. Acta Psychiatr Scand 1983 Jun;67(6):361-370.

(19) Lacey RJ, Strauss VY, Rathod T, Belcher J, Croft PR, Natvig B, et al. Clustering of pain and its associations with health in people aged 50 years and older: cross-sectional results from the North Staffordshire Osteoarthritis Project. BMJ Open 2015 November 01;5(11).

(20) Jones BL, Nagin DS. Advances in Group-Based Trajectory Modeling and an SAS Procedure for Estimating Them. Sociological Methods & Research 2007 May 01;35(4):542-571.

(21) Clark DB, Jones BL, Wood DS, Cornelius JR. Substance use disorder trajectory classes: diachronic integration of onset age, severity, and course. Addict Behav 2006 Jun;31(6):995-1009.

(22) Clark AL, Goode KM. Do patients with chronic heart failure have chest pain? Int J Cardiol 2013 Jul 15;167(1):185-189.

(23) Ramasamy R, Hildebrandt T, O'Hea E, Patel M, Clemow L, Freudenberger R, et al. Psychological and Social Factors That Correlate With Dyspnea in Heart Failure. Psychosomatics 2006 0;47(5):430-434.

For peer review only - http://bmjopen.bmj.com/site/about/guidelines.xhtml

(24) Fan X, Meng Z. The mutual association between depressive symptoms and dyspnea in Chinese patients with chronic heart failure. European Journal of Cardiovascular Nursing 2015 August 01;14(4):310-316.

(25) Hayek SS, Ko Y, Awad M, Del Mar Soto A, Ahmed H, Patel K, et al. Depression and chest pain in patients with coronary artery disease. Int J Cardiol 2017 3/1;230:420-426.

(26) Jespersen L, Abildstrom SZ, Hvelplund A, Prescott E. Persistent angina: highly prevalent and associated with long-term anxiety, depression, low physical functioning, and quality of life in stable angina pectoris. Clin Res Cardiol 2013 Aug;102(8):571-581.

(27) Bernhardt V, Babb TG. Weight loss reduces dyspnea on exertion in obese women. Respir Physiol Neurobiol 2014 Dec 1;204:86-92.

(28) Abidov A, Rozanski A, Hachamovitch R, Hayes SW, Aboul-Enein F, Cohen I, et al. Prognostic Significance of Dyspnea in Patients Referred for Cardiac Stress Testing. N Engl J Med 2005 11/03; 2017/02;353(18):1889-1898.

(29) Jordan K, Jöud A, Bergknut C, Croft P, Edwards J, Peat G, et al. International comparisons of the consultation prevalence of musculoskeletal conditions using population-based health care data from England and Sweden. Ahn Rheum Dis. 2013;73(1):212-218.

For peer review only

For peer review only - http://bmjopen.bmj.com/site/about/guidelines.xhtml

Table 1: Sample characteristics, *n* (%)

	Invited at baseline	Baseline responders	At least 1 monthly response	At least 2 monthly responses[a]	At least 5 monthly responses[b]
n	2858	1696	871	777	661
Condition Group:					
IHD	2526 (88)	1501 (89)	783 (90)	701 (90)	603 (91)
HF	332 (12)	195 (11)	88 (10)	76 (10)	58 (9)
Age:					
<60	401 (14)	172 (10)	103 (12)	89 (11)	75 (11)
60-69	782 (27)	463 (27)	265 (30)	241 (31)	201 (30)
70-79	987 (35)	652 (38)	339 (39)	304 (39)	274 (41)
80+	688 (24)	409 (24)	164 (19)	143 (18)	111 (17)
Gender:					
Male	1712 (60)	1059 (62)	574 (66)	514 (66)	447 (68)
Female	1146 (40)	637 (38)	297 (34)	263 (34)	214 (32)
Baseline chest pain:	N/A	689 (44)	359 (44)	318 (43)	266 (42)
Baseline SOB:	N/A	942 (58)	495 (58)	430 (57)	354 (54)

[a] population for sensitivity analysis; [b] population for main analysis.

SOB: shortness of breath, IHD: ischaemic heart disease, HF: heart failure

For peer review only

Table 2: Associations between baseline characteristics and monthly chest pain (n=661)

Baseline characteristics	Monthly chest pain			
	1-3 weeks *vs.* none		4 weeks *vs.* none	
	Unadjusted RRR (95% CI)	Adjusted[a] RRR (95% CI)	Unadjusted RRR (95% CI)	Adjusted[a] RRR (95% CI)
Male	1	1	1	1
Female	**1.50 (1.08, 2.08)**	1.20 (0.77, 1.54)	1.48 (0.90, 2.43)	1.29 (0.73, 2.28)
Age				
<60	1	1	1	1
60-69	1.35 (0.81, 2.23)	1.23 (0.74, 2.06)	0.64 (0.31, 1.33)	0.65 (0.28, 1.49)
70-79	1.39 (0.85, 2.27)	1.41 (0.86, 2.34)	0.49 (0.24, 0.99)	0.49 (0.21, 1.11)
80+	1.25 (0.68, 2.29)	1.18 (0.64, 2.16)	0.42 (0.17, 1.03)	0.47 (0.17, 1.28)
BMI				
Normal	1	1	1	1
Overweight	0.88 (0.60, 1.28)	0.79 (0.54, 1.15)	0.93 (0.53, 1.65)	0.80 (0.42, 1.51)
Obese	0.95 (0.63, 1.45)	0.67 (0.43, 1.03)	1.77 (0.94, 3.34)	0.74 (0.36, 1.54)
PCS score (per unit)	**0.95 (0.93, 0.96)**	-	**0.89 (0.87, 0.91)**	-
Not anxious or depressed	1	1	1	1
Anxious or depressed	**2.55 (1.85, 3.51)**	**2.32 (1.66, 3.24)**	**5.42 (3.19, 9.19)**	**3.31 (1.89, 5.79)**
Pain sites:				
None	1	1	1	1
1-3	**3.38 (1.97, 5.81)**	**3.01 (1.77, 5.13)**	1.19 (0.50, 2.81)	1.24 (0.51, 3.01)
4+	**5.66 (3.32, 9.66)**	**3.89 (2.25, 6.74)**	**6.16 (2.69, 14.14)**	**4.03 (1.64, 9.91)**
Between person Variance	-	2.39	-	7.07

[a] adjusted for all presented variables. RRR: relative risk ratio, CI: confidence interval, BMI: body mass index, PCS: physical component summary score. **Bold** = statistically significant

For peer review only - http://bmjopen.bmj.com/site/about/guidelines.xhtml

Table 3: Associations between baseline characteristics and monthly shortness of breath (n=661)

Baseline characteristics	Monthly shortness of breath			
	1-3 weeks *vs.* none		4 weeks *vs.* none	
	Unadjusted RRR (95% CI)	Adjusted[a] RRR (95% CI)	Unadjusted RRR (95% CI)	Adjusted[a] RRR (95% CI)
Male	1	1	1	1
Female	**1.84 (1.36, 2.49)**	**1.46 (1.07, 2.01)**	**2.58 (1.78, 3.75)**	**1.81 (1.20, 2.75)**
Age				
<60	1	1	1	1
60-69	1.30 (0.81, 2.07)	1.18 (0.74, 1.90)	1.10 (0.62, 1.95)	0.92 (0.49, 1.71)
70-79	**1.80 (1.14, 2.83)**	**1.83 (1.15, 2.90)**	1.40 (0.80, 2.44)	1.72 (0.94, 3.17)
80+	**1.91 (1.10, 3.31)**	**2.47 (1.41, 4.34)**	1.53 (0.77, 3.02)	**2.51 (1.19, 5.26)**
BMI				
Normal	1	1	1	1
Overweight	1.25 (0.88, 1.76)	1.28 (0.90, 1.81)	1.02 (0.66, 1.58)	1.03 (0.64, 1.65)
Obese	**1.77 (1.20, 2.62)**	**1.67 (1.12, 2.51)**	**2.93 (1.80, 4.78)**	**2.53 (1.49, 4.30)**
PCS score (per unit)	**0.93 (0.92, 0.94)**	-	**0.87 (0.86, 0.89)**	-
Not anxious or depressed	1	1	1	1
Anxious or depressed	**3.04 (2.26, 4.08)**	**2.33 (1.70, 3.19)**	**5.36 (3.67, 7.82)**	**4.10 (2.72, 6.17)**
Pain sites:				
None	1	1	1	1
1-3	**2.23 (1.41, 3.54)**	**1.98 (1.23, 3.17)**	**4.06 (2.20, 7.50)**	**3.50 (1.80, 6.80)**
4+	**4.31 (2.74, 6.80)**	**3.05 (1.88, 4.97)**	**8.90 (4.87, 16.29)**	**4.64 (2.35,9.18)**
Between person Variance	-	2.09	-	3.72

[a] adjusted for all presented variables. RRR: relative risk ratio, CI: confidence interval, BMI: body mass index, PCS: physical component summary score. **Bold** = statistically significant

For peer review only - http://bmjopen.bmj.com/site/about/guidelines.xhtml

Table 4 : Goodness of fit of the dual trajectory models with differing numbers of clusters

Clusters	AIC	BIC	Adjusted BIC	Smallest class (overall)	AvePP[a] (range)
1	23241.43	23277.38	23251.98		
2	17999.11	18066.52	18018.89		
3	16185.44	16284.30	16214.45	16%	0.98-0.98
4	15088.50	15218.82	15126.74	15%	0.95-0.99
5	14310.02	14471.80	14357.50	9%	0.94-0.99
6	14018.94	14212.17	14075.64	7%	0.93-0.98
7	13820.48	14045.17	13886.42	4%	0.92-0.98
8	13701.00	13957.15	13776.17	3%	0.89-0.99
9	13628.19	13915.79	13712.59	0.1%	0.88-1.00

[a] Average posterior probability for participants allocated to cluster. AIC: Akaike information criterion, BIC: Bayesian information criterion.

For peer review only

Table 5: Summary of the clusters (8-cluster model)

Description	Cluster 1 No Symptoms	Cluster 2 Infrequent SOB	Cluster 3 Occasional chest pain	Cluster 4 Occasional SOB	Cluster 5 Occasional chest pain & SOB	Cluster 6 Frequent chest pain & SOB	Cluster 7 Persistent SOB	Cluster 8 Persistent chest pain & SOB
Number of patients in cluster (%)	166 (25)	93 (14)	20 (3)	83 (13)	125 (19)	75 (11)	52 (8)	47 (7)
**Range of monthly probabilities of extent of chest pain / SOB**								
Chest pain								
None	0.95-1.00	0.86-0.91	0.13-0.25	0.85-0.91	0.22-0.27	0.06-0.10	0.85-0.92	<0.01
Up to 3 weeks	<0.01-0.05	0.09-0.14	0.66-0.68	0.09-0.15	0.64-0.67	0.57-0.66	0.08-0.15	0.04-0.10
4 weeks	<0.01	<0.01	0.10-0.20	<0.01	0.09-0.11	0.24-0.37	<0.01	0.90-0.96
SoB								
None	0.96-0.99	0.52-0.65	0.76-0.93	0.13-0.19	0.15-0.16	0.01-0.02	<0.01	<0.01
Up to 3 weeks	0.01-0.04	0.34-0.46	0.07-0.23	0.72-0.74	0.73-0.73	0.33-0.45	0.11-0.16	0.03-0.12
4 weeks	<0.01	0.01-0.02	<0.01	0.09-0.13	0.11-0.12	0.53-0.66	0.84-0.88	0.87-0.97
**Median (IQR) % of months with chest pain / SOB**								
Chest pain								
None	100 (100,100)	90 (80,100)	21 (0,50)	100 (80,100)	20 (0,40)	0 (0,14)	100 (81,100)	0 (0,0)
Up to 3 weeks	0 (0,0)	10 (0,20)	60 (40,79)	0 (0,20)	70 (56,89)	57 (40,70)	0 (0,13)	0 (0,13)
4 weeks	0 (0,0)	0 (0,0)	0 (0,16)	0 (0,0)	0 (0,11)	33 (17,50)	0 (0,0)	100 (83,100)
SoB								
None	100 (100,100)	63 (50,75)	100 (79,100)	11 (0,25)	10 (0,20)	0 (0,0)	0 (0,0)	0 (0,0)
Up to 3 weeks	0 (0,0)	30 (20,44)	0 (0,17)	80 (70,90)	80 (63,100)	33 (20,50)	0 (0,20)	0 (0,13)
4 weeks	0 (0,0)	0 (0,0)	0 (0,0)	0 (0,20)	0 (0,11)	60 (50,78)	90 (79,100)	100 (86,100)

SoB: shortness of breath

For peer review only - http://bmjopen.bmj.com/site/about/guidelines.xhtml

Table 6: Comparison between clusters on baseline socio-demographic and general health measures, *n* (%) unless otherwise stated

Description	Cluster 1 No symptoms	Cluster 2 Infrequent SOB	Cluster 3 Occasional chest pain	Cluster 4 Occasional SOB	Cluster 5 Occasional chest pain & SOB	Cluster 6 Frequent chest pain & SOB	Cluster 7 Persistent SOB	Cluster 8 Persistent chest pain & SOB
Number of patients in cluster	166 (25)	93 (14)	20 (3)	83 (13)	125 (19)	75 (11)	52 (8)	47 (7)
Female	33 (20)	31 (33)	5 (25)	29 (35)	43 (34)	31 (41)	22 (42)	20 (43)
Age								
<60	26 (16)	7 (8)	4 (20)	9 (11)	11 (9)	6 (8)	2 (4)	10 (21)
60-69	59 (36)	28 (30)	5 (25)	17 (20)	42 (34)	17 (23)	17 (33)	16 (34)
70-79	59 (36)	40 (43)	9 (45)	37 (45)	53 (42)	34 (45)	24 (46)	18 (38)
80+	22 (13)	18 (19)	2 (10)	20 (24)	19 (15)	18 (24)	9 (17)	3 (6)
BMI group								
Normal weight	50 (31)	24 (28)	10 (50)	16 (20)	30 (26)	17 (25)	10 (20)	9 (20)
Overweight	81 (50)	47 (55)	7 (35)	39 (49)	57 (49)	29 (43)	16 (31)	14 (32)
Obese	31 (19)	15 (17)	3 (15)	25 (31)	29 (25)	22 (32)	25 (49)	21 (48)
Mean PCS (SD)	47.1 (10.26)	39.5 (10.89)	39.8 (11.4)	38.3 (10.43)	36.93 (9.71)	28.5 (8.91)	31.0 (9.99)	24.6 (7.05)
Anxious or depressed	17 (11)	20 (22)	6 (30)	32 (40)	51 (42)	48 (69)	20 (38)	29 (63)
Pain sites								
None	38 (23)	15 (16)	2 (10)	5 (6)	8 (6)	3 (4)	4 (8)	1 (2)
1-3 sites	83 (50)	39 (42)	8 (40)	30 (36)	45 (36)	19 (25)	25 (48)	10 (21)
4 plus sites	45 (27)	39 (42)	10 (50)	48 (58)	72 (58)	53 (71)	23 (44)	36 (77)

SOB: shortness of breath, BMI: body mass index, PCS: physical component summary score, SD: standard deviation

For peer review only

Supplementary Table 1: Results from sensitivity analysis of associations with monthly chest pain using respondents that answered 2 or more monthly questionnaires

	1-3 weeks vs none		4 weeks vs none	
Chest pain (*n* = 777)	Unadjusted RRR (95% CI)	Adjusted[a] RRR (95% CI)	Unadjusted RRR (95% CI)	Adjusted[a] RRR (95% CI)
Age: <60	1	1	1	1
60-69	1.37 (0.86, 2.19)	1.23 (0.76, 1.99)	0.70 (0.35, 1.39)	0.67(0.31, 1.46)
70-79	1.48 (0.93, 2.33)	1.43 (0.89, 2.29)	0.52 (0.26, 1.02)	0.52 (0.24, 1.14)
80+	1.26 (0.73, 2.18)	1.26 (0.72, 2.22)	0.38 (0.17, 0.88)	0.45 (0.17, 1.16)
BMI: Normal	1	1	1	1
Overweight	0.84 (0.59, 1.18)	0.76 (0.54, 1.09)	0.98 (0.56, 1.69)	0.78 (0.42, 1.43)
Obese	0.91 (0.62, 1.33)	0.64 (0.42, 0.95)	1.68 (0.92, 3.05)	0.69 (0.35, 1.39)
Male	1	1	1	1
Female	1.44 (1.07, 1.94)	1.16 (0.84, 1.58)	1.29 (0.81, 2.07)	1.17 (0.68, 2.01)
PCS score (per unit)	0.95 (0.94, 0.96)	-	0.90 (0.88, 0.92)	-
Not anxious/depressed	1	1	1	1
Anxious or depressed	2.66 (1.99, 3.56)	2.33 (1.71, 3.17)	4.84 (2.97, 7.89)	3.20 (1.89, 5.40)
Pain sites: None	1	1	1	1
1-3	3.73 (2.25, 6.21)	3.25 (1.95, 5.41)	1.45 (0.64, 3.29)	1.36 (0.57, 3.22)
4+	6.26 (3.79, 10.33)	4.36 (2.58, 7.38)	6.23 (2.82, 13.78)	4.21 (1.76, 10.11)
Between person variance	-	2.29	-	6.99

For peer review only

Supplementary Table 2: Results from sensitivity analysis of associations with monthly SOB using respondents that answered 2 or more monthly questionnaires.

		1-3 weeks vs none		4 weeks vs none	
SoB (*n* = 777)		Unadjusted RRR (95% CI)	Adjusted[a] RRR (95% CI)	Unadjusted RRR (95% CI)	Adjusted[a] RRR (95% CI)
Age:	<60	1	1	1	1
	60-69	1.19 (0.77, 1.83)	1.06 (0.69, 1.65)	1.03 (0.60, 1.74)	0.98 (0.56, 1.72)
	70-79	1.62 (1.06, 2.47)	1.75 (1.13, 2.69)	1.24 (0.74, 2.09)	1.73 (0.99, 3.02)
	80+	1.88 (1.13, 3.12)	2.25 (1.34, 3.76)	1.43 (0.77, 2.67)	2.36 (1.22, 4.60)
BMI:	Normal	1	1	1	1
	Overweight	1.12 (0.81, 1.55)	1.17 (0.84, 1.62)	0.99 (0.66, 1.49)	0.96 (0.62, 1.47)
	Obese	1.71 (1.20, 2.45)	1.53 (1.05, 2.22)	2.86 (1.83, 4.48)	2.20 (1.36, 3.54)
Male		1	1	1	1
Female		1.91 (1.45, 2.52)	1.42 (1.06, 1.90)	2.31 (1.64, 3.25)	1.62 (1.11, 2.34)
PCS score (per unit)		0.93 (0.92, 0.94)	-	0.87 (0.86, 0.89)	-
Not anxious/depressed		1	1	1	1
Anxious or depressed		3.09 (2.36, 4.06)	2.46 (1.85, 3.29)	5.38 (3.81, 7.59)	3.84 (2.66, 5.55)
Pain sites:	None	1	1	1	1
	1-3	2.47 (1.59, 3.84)	2.28 (1.46, 3.55)	4.47 (2.48, 8.05)	3.84 (2.07, 7.14)
	4+	5.02 (3.25, 7.74)	3.51 (2.22, 5.56)	10.46 (5.87, 18.63)	5.60 (2.97, 10.56)
Between person variance		-	2.02	-	3.46

For peer review only

Supplementary Table 3**:** Results from multiple imputation analysis of associations with monthly chest pain response

Multiple Imputation	Monthly chest pain			
Baseline characteristics	1-3 weeks *vs.* none		4 weeks *vs.* none	
	Unadjusted RRR (95% CI)	Adjusted[a] RRR (95% CI)	Unadjusted RRR (95% CI)	Adjusted[a] RRR (95% CI)
Age				
<60	1	1	1	1
60-69	1.27 (0.83, 1.94)	1.22 (0.80, 1.87)	0.66 (0.37, 1.19)	0.68 (0.36, 1.31)
70-79	1.40 (0.94, 2.11)	1.34 (0.89, 2.02)	0.59 (0.33, 1.05)	0.60 (0.32, 1.13)
80+	1.32 (0.83, 2.10)	1.31 (0.81, 2.11)	0.44 (0.22, 0.86)	0.49 (0.23, 1.04)
BMI				
Normal	1	1	1	1
Overweight	0.94 (0.70, 1.27)	0.94 (0.69, 1.27)	0.97 (0.64, 1.49)	0.90 (0.57, 1.43)
Obese	0.94 (0.69, 1.28)	0.79 (0.58, 1.10)	1.71 (1.10, 2.65)	1.17 (0.72, 1.91)
Gender				
Male	1	1	1	1
Female	**1.52 (1.18, 1.96)**	1.27 (0.97, 1.67)	1.43 (0.98, 2.08)	1.20 (0.78, 1.85)
Anxiety/depression				
Not anxious or depressed	1	1	1	1
Anxious or depressed	**2.92 (2.28, 3.74)**	**2.48 (1.84, 3.35)**	**4.69 (3.17, 6.94)**	**3.14 (2.09, 4.71)**
Pain sites:				
None	1	1	1	1
1-3	**3.01 (2.01, 4.54)**	**2.87 (1.90, 4.33)**	1.77 (0.81, 3.88)	1.53 (0.71, 3.31)
4+	**4.43 (3.03, 6.48)**	**3.53 (2.37, 5.26)**	**5.89 (3.20, 10.85)**	**3.91 (1.99, 7.68)**
Between person variance	-	6.07	-	128.51

Supplementary Table 4: Results from multiple imputation analysis of associations with monthly SoB response

Multiple Imputation	Monthly SOB			
Baseline characteristics	1-3 weeks *vs.* none		4 weeks *vs.* none	
	Unadjusted RRR (95% CI)	Adjusted[a] RRR (95% CI)	Unadjusted RRR (95% CI)	Adjusted[a] RRR (95% CI)
Age				
<60	1	1	1	1
60-69	1.37 (0.92, 2.05)	1.36 (0.90, 2.06)	1.05 (0.65, 1.69)	1.10 (0.66, 1.82)
70-79	1.79 (1.22, 2.63)	1.91 (1.29, 2.83)	1.34 (0.85, 2.12)	1.65 (1.01, 2.69)
80+	1.92 (1.25, 2.96)	2.33 (1.50, 3.61)	1.36 (0.80, 2.30)	2.12 (1.21, 3.70)
BMI				
Normal	1	1	1	1
Overweight	1.24 (0.95, 1.62)	1.32 (1.00, 1.74)	1.02 (0.74, 1.41)	1.09 (0.77, 1.54)
Obese	1.51 (1.11, 2.06)	1.56 (1.11, 2.20)	2.61 (1.84, 3.70)	2.60 (1.77, 3.82)
Gender				
Male	1	1	**1**	1
Female	**1.75 (1.38, 2.22)**	**1.40 (1.09, 1.80)**	**2.15 (1.61, 2.88)**	1.59 (1.16, 2.18)
Anxiety/depression				
Not anxious or depressed	1	1	1	1
Anxious or depressed	**2.93 (2.25, 3.82)**	**2.49 (1.88, 3.31)**	**5.16 (3.84, 6.94)**	**4.05 (2.89, 5.68)**
Pain sites:				
None	1	1	1	1
1-3	**2.17 (1.52, 3.10)**	**1.99 (1.36, 2.90)**	3.77 (2.24, 6.35)	3.09 (1.78, 5.36)
4+	**3.38 (2.35, 4.86)**	**2.61 (1.75, 3.90)**	6.44 (3.91, 10.63)	**3.93 (2.24, 6.89)**
Between person variance	-	5.08	-	14.80

For peer review only

Supplementary Table 5: Summary of the clusters (8-cluster model) including patients responding to at least 2 monthly questionnaires

Description	Cluster 1 No symptoms	Cluster 2 Infrequent SOB	Cluster 3 Occasional pain	Cluster 4 Occasional SOB	Cluster 5 Occasional pain & SOB	Cluster 6 Frequent pain & SOB	Cluster 7 Persistent SOB	Cluster 8 Persistent pain & SOB
Observed *n* (%)	189 (24)	106 (14)	20 (3)	89 (11)	161 (21)	96 (12)	66 (8)	50 (6)
Range of monthly probabilities of extent of chest pain / SOB								
Chest pain None	0.95-1.00	0.85-0.90	0.14-0.24	0.85-0.91	0.22-0.25	0.06-0.11	0.83-0.93	<0.01
Up to 3 weeks	<0.01-0.05	0.10-0.14	0.66-0.68	0.09-0.14	0.66-0.68	0.58-0.68	0.07-0.16	0.04-0.11
4 weeks	<0.01	<0.01	0.10-0.20	<0.01	0.09-0.11	0.21-0.36	<0.01	0.89-0.96
SOB None	0.96-0.99	0.51-0.65	0.76-0.93	0.13-0.18	0.14-0.15	0.01-0.02	<0.01	<0.01
Up to 3 weeks	0.01-0.04	0.33-0.47	0.07-0.23	0.73-0.74	0.73-0.74	0.33-0.43	0.11-0.16	0.03-0.11
4 weeks	<0.01	0.01-0.02	<0.01	0.10-0.13	0.11-0.13	0.55-0.66	0.83-0.87	0.88-0.96

SOB: shortness of breath

For peer review only

Supplementary Table 6: Comparison between clusters on baseline socio-demographic and general health measures using multiple imputed data (m=50)

Description	Cluster 1 No symptoms	Cluster 2 Infrequent SOB	Cluster 3 Occasional pain	Cluster 4 Occasional SOB	Cluster 5 Occasional pain & SOB	Cluster 6 Frequent pain & SOB	Cluster 7 Persistent SOB	Cluster 8 Persistent pain & SOB
Observed %	22	14	3	12	18	14	9	8
Female *n* (%)	24	40	30	42	38	43	50	41
Age <60	13	7	21	8	10	6	5	20
60-69	33	26	29	23	29	21	25	32
70-79	37	39	37	40	39	39	41	35
80+	17	28	14	30	22	34	30	13
BMI group								
Normal weight	31	28	51	22	29	29	20	23
Overweight	50	53	34	47	46	40	30	32
Obese	19	18	15	31	25	31	49	44
PCS mean	45.5 (43.9, 47.1)	37.7 (35.6, 39.7)	38.9 (34.8, 42.9)	36.7 (34.6, 38.7)	35.7 (34.0, 37.3)	27.6 (25.9, 29.3)	29.8 (27.4, 32.2)	25.0 (22.8, 27.2)
Pain sites								
None	26	17	13	8	7	5	12	3
1-3 sites	49	44	44	37	35	27	45	20
4 plus sites	25	39	43	55	58	68	43	76
Anxious or depressed	16	35	38	49	52	77	50	73

SOB: shortness of breath. 50 imputations: model included baseline PCS score, anxiety, depression chest pain, SOB, cluster, age, gender, heart failure, number of pain sites

For peer review only

STROBE Statement—checklist of items that should be included in reports of observational studies

	Item No	Recommendation
**Title and abstract**	1	(*a*) Indicate the study's design with a commonly used term in the title or the abstract **[within the title page 1]**
		(*b*) Provide in the abstract an informative and balanced summary of what was done and what was found **[within the abstract: page 2]**
**Introduction**		
Background/rationale	2	Explain the scientific background and rationale for the investigation being reported **[page 3-4]**
Objectives	3	State specific objectives, including any prespecified hypotheses **[pages 4]**
**Methods**		
Study design	4	Present key elements of study design early in the paper **[p 4]**
Setting	5	Describe the setting, locations, and relevant dates, including periods of recruitment, exposure, follow-up, and data collection **[page 5]**
Participants	6	(*a*) *Cohort study*—Give the eligibility criteria, and the sources and methods of selection of participants. Describe methods of follow-up **[pages 5]**<br>*Case-control study*—Give the eligibility criteria, and the sources and methods of case ascertainment and control selection. Give the rationale for the choice of cases and controls<br>*Cross-sectional study*—Give the eligibility criteria, and the sources and methods of selection of participants
		(*b*) *Cohort study*—For matched studies, give matching criteria and number of exposed and unexposed<br>*Case-control study*—For matched studies, give matching criteria and the number of controls per case
Variables	7	Clearly define all outcomes, exposures, predictors, potential confounders, and effect modifiers. Give diagnostic criteria, if applicable **[pages 5-6]**
Data sources/ measurement	8*	For each variable of interest, give sources of data and details of methods of assessment (measurement). Describe comparability of assessment methods if there is more than one group **[pages 5-6]**
Bias	9	Describe any efforts to address potential sources of bias **[N/A]**
Study size	10	Explain how the study size was arrived at **[pages 7-8]**
Quantitative variables	11	Explain how quantitative variables were handled in the analyses. If applicable, describe which groupings were chosen and why **[pages 7-9]**
Statistical methods	12	(*a*) Describe all statistical methods, including those used to control for confounding **[pages 7-9]**
		(*b*) Describe any methods used to examine subgroups and interactions **[pages 7-9]**
		(*c*) Explain how missing data were addressed **[page 7 & 8]**
		(*d*) *Cohort study*—If applicable, explain how loss to follow-up was addressed **[page 8]**<br>*Case-control study*—If applicable, explain how matching of cases and controls was addressed<br>*Cross-sectional study*—If applicable, describe analytical methods taking account of sampling strategy
		(*e*) Describe any sensitivity analyses **[page 7 & 8]**

For peer review only

Continued on next page

For peer review only

For peer review only - http://bmjopen.bmj.com/site/about/guidelines.xhtml

**Results**		
Participants	13*	(a) Report numbers of individuals at each stage of study—eg numbers potentially eligible, examined for eligibility, confirmed eligible, included in the study, completing follow-up, and analysed **[pages 9-10]**
		(b) Give reasons for non-participation at each stage **[N/A]**
		(c) Consider use of a flow diagram **[N/A]**
Descriptive data	14*	(a) Give characteristics of study participants (eg demographic, clinical, social) and information on exposures and potential confounders **[pages 9-10]**
		(b) Indicate number of participants with missing data for each variable of interest **[N/A]**
		(c) *Cohort study*—Summarise follow-up time (eg, average and total amount) **[page 9]**
Outcome data	15*	*Cohort study*—Report numbers of outcome events or summary measures over time **[pages 9-10]**
		Case-control study—Report numbers in each exposure category, or summary measures of exposure
		Cross-sectional study—Report numbers of outcome events or summary measures
Main results	16	(*a*) Give unadjusted estimates and, if applicable, confounder-adjusted estimates and their precision (eg, 95% confidence interval). Make clear which confounders were adjusted for and why they were included **[pages 9-11]**
		(*b*) Report category boundaries when continuous variables were categorized **[pages 9-11]**
		(*c*) If relevant, consider translating estimates of relative risk into absolute risk for a meaningful time period **[N/A]**
Other analyses	17	Report other analyses done—eg analyses of subgroups and interactions, and sensitivity analyses **[pages 9-11]**
**Discussion**		
Key results	18	Summarise key results with reference to study objectives **[pages 15]**
Limitations	19	Discuss limitations of the study, taking into account sources of potential bias or imprecision. Discuss both direction and magnitude of any potential bias **[pages 14-15]**
Interpretation	20	Give a cautious overall interpretation of results considering objectives, limitations, multiplicity of analyses, results from similar studies, and other relevant evidence **[page 12-14]**
Generalisability	21	Discuss the generalisability (external validity) of the study results **[pages 12-14]**
**Other information**		
Funding	22	Give the source of funding and the role of the funders for the present study and, if applicable, for the original study on which the present article is based **[page 16]**

*Give information separately for cases and controls in case-control studies and, if applicable, for exposed and unexposed groups in cohort and cross-sectional studies.

Note: An Explanation and Elaboration article discusses each checklist item and gives methodological background and published examples of transparent reporting. The STROBE checklist is best used in conjunction with this article (freely available on the Web sites of PLoS Medicine at http://www.plosmedicine.org/, Annals of Internal Medicine at http://www.annals.org/, and Epidemiology at http://www.epidem.com/). Information on the STROBE Initiative is available at www.strobe-statement.org.

For peer review only

For peer review only - http://bmjopen.bmj.com/site/about/guidelines.xhtml
